# Association of DNA Methylation in Blood Pressure-Related Genes With Ischemic Stroke Risk and Prognosis

**DOI:** 10.3389/fcvm.2022.796245

**Published:** 2022-03-08

**Authors:** Huan Zhang, Xingbo Mo, Aili Wang, Hao Peng, Daoxia Guo, Chongke Zhong, Zhengbao Zhu, Tan Xu, Yonghong Zhang

**Affiliations:** ^1^Jiangsu Key Laboratory of Preventive and Translational Medicine for Geriatric Diseases, Medical College of Soochow University, Suzhou, China; ^2^Department of Epidemiology, School of Public Health, Medical College of Soochow University, Suzhou, China; ^3^Center for Genetic Epidemiology and Genomics, School of Public Health, Medical College of Soochow University, Suzhou, China

**Keywords:** prognosis, methylation, stroke, blood pressure, mortality

## Abstract

**Background:**

A genome-wide association study identified 12 genetic loci influencing blood pressure and implicated a role of DNA methylation. However, the relationship between methylation and ischemic stroke has not yet been clarified. We conducted a large-sample sequencing study to identify blood leukocyte DNA methylations as novel biomarkers for ischemic stroke risk and prognosis based on previously identified genetic loci.

**Methods:**

Methylation levels of 17 genes were measured by sequencing in 271 ischemic stroke cases and 323 controls, and the significant associations were validated in another independent sample of 852 cases and 925 controls. The associations between methylation levels and ischemic stroke risk and prognosis were evaluated.

**Results:**

Methylation of *AMH, C17orf82, HDAC9, IGFBP3, LRRC10B, PDE3A, PRDM6, SYT7* and *TBX2* was significantly associated with ischemic stroke. Compared to participants without any hypomethylated targets, the odds ratio (OR) (95% confidence interval, CI) for those with 9 hypomethylated genes was 1.41 (1.33–1.51) for ischemic stroke. Adding methylation levels of the 9 genes to the basic model of traditional risk factors significantly improved the risk stratification for ischemic stroke. Associations between *AMH, HDAC9, IGFBP3, PDE3A* and *PRDM6* gene methylation and modified Rankin Scale scores were significant after adjustment for covariates. Lower methylation levels of *AMH, C17orf82, PRDM6* and *TBX2* were significantly associated with increased 3-month mortality. Compared to patients without any hypomethylated targets, the OR (95% CI) for those with 4 hypomethylated targets was 1.12 (1.08–1.15) for 3-month mortality (*P* = 2.28 × 10^−10^).

**Conclusion:**

The present study identified blood leukocyte DNA methylations as potential factors affecting ischemic stroke risk and prognosis among Han Chinese individuals.

## Introduction

Hypertension is one of the most important risk factors for ischemic stroke and the second leading cause of death worldwide (1, 2). The prevalence has risen in recent decades, and our ability to predict mortality and functional outcomes after acute ischemic stroke remains poor. As complex diseases, genetic and epigenetic factors play important roles in the pathogenesis of ischemic stroke.

Genome-wide association studies (GWASs) have identified numerous genetic loci for blood pressure (BP) and ischemic stroke (3–5). A trans-ancestry GWAS identified 12 genetic loci influencing BP (6). Sequence variants in these genes have a direct effect on DNA methylation levels in leukocytes; moreover, methylation in some of these genes was associated with BP. However, knowledge about the contribution of the identified genetic components to ischemic stroke is vague at best. The sentinel SNPs in these 12 loci pointed to 20 candidate genes, including several genes involved in vascular smooth muscle (*IGFBP3, KCNK3, PDE3A* and *PRDM6*) (7–10) and renal (*ARHGAP24, OSR1, SLC22A7* and *TBX2*) (11–14) function. Genetic variations in some genes (e.g., *SLC22A7, ZNF318* and *HDAC9*) were also associated with ischemic stroke (5). In addition, *IGFBP3* and *HDAC9* have been found to be related to poor prognosis in patients with ischemic stroke (15–17). Therefore, the identified BP-related loci may contain genes involved in ischemic stroke.

DNA methylation, which occurs primarily on CpG dinucleotides to control transcription, is a key player in diseases (18, 19). DNA methylation has been identified as a potential drug target in the treatment of stroke (20) and could be used as a potential predictor of stroke risk (21) and prognosis (22–25). Studies have also suggested that heritable DNA methylation plays a role in regulating BP (26), and DNA methylation associated with BP was also associated with stroke (27). Even so, known DNA methylation for stroke is limited. Identification of novel DNA methylation for ischemic stroke will uncover new biomarkers and increase our understanding of the pathogenic molecular mechanism.

Although the associations of genetic variants of the 20 genes and methylation in some of these genes with BP have been established, until now, the relationship between methylation of these genes and ischemic stroke has not yet been clarified. If DNA methylation in these genes was strongly associated with BP, they might have an impact on ischemic stroke risk. Therefore, we conducted a two-stage design case-control study to investigate the associations between DNA methylation in these genes and ischemic stroke. In addition, we examined the associations between methylation levels of these genes and 3-month outcome in acute ischemic stroke patients by integrating clinical data from the China Antihypertensive Trial in Acute Ischemic Stroke (CATIS) project (28).

## Methods

### Study Sample

Ischemic stroke cases were randomly selected from the China Antihypertensive Trial in Acute Ischemic Stroke (CATIS) project—a multicenter, single-blind, blinded end point, randomized clinical trial conducted in 26 hospitals across China from August 2009 to May 2014 (28). In brief, 4,071 patients aged 22 years or older who had ischemic stroke confirmed by computed tomography or magnetic resonance imaging of the brain within 48 h of symptom onset and who had an elevated systolic BP (SBP) between 140 and 220 mmHg were recruited. The study design, intervention and survey methods of CATIS have been described previously (28).

Sex- and age-matched controls were selected from the Suzhou Metabolic Syndrome Study (SMSS) (6). SMSS was an observational cohort study on the relationship between metabolic syndrome and its components and cardiovascular disease events in 6 rural townships in Suzhou, China. A total of 18,461 individuals who had no evidence of end organ damage, including coronary heart disease, stroke, chronic renal disease and tumors, and signed informed consent were recruited to participate in the SMSS. Among these participants selected as the control sample, participants with hypertension served as hypertensive controls, and those without hypertension served as normotensive controls. By considering both sequencing cost and statistical power, 271 IS cases and 323 controls in stage one and 852 cases and 925 controls in stage two were chosen from the cohorts and successfully sequenced in this study.

### Standard Protocol Approvals, Registrations, and Patient Consents

The CATIS project was registered at clinicaltrials.gov (identifier: NCT01840072). The study protocols were approved by the institutional review boards or ethical committees at Soochow University. Written consent was obtained from all study participants or their immediate family members.

### Data Collection

For all the participants, data on demographic information, lifestyle risk factors, family history of cardiovascular disease, and personal medical history were obtained using a standard questionnaire administered by trained staff. Three consecutive sitting BP measurements (30 s between each) were taken by trained staff using a standard mercury sphygmomanometer according to a standard protocol after the subjects had been resting for 30 min. The first and fifth Korotkoff sounds were recorded as the SBP and DBP, respectively. The mean of the three readings was used in the analysis. Hypertension was defined as SBP ≥140 mmHg and/or DBP ≥90 mmHg and/or the use of antihypertensive medication in the last 2 weeks. The time of stroke onset was recorded for each patient. Stroke severity was assessed with the NIH Stroke Scale (NIHSS) by trained neurologists at admission (29). Participants were followed up in person at 3 months by trained neurologists unaware of treatment assignment. A combination of death and major disability [modified Rankin Scale (mRS) score ≥3] was used to measure stroke prognosis. Scores on the mRS range from 0 to 6, with a score of 0 indicating no symptoms, a score of 5 indicating severe disability (i.e., bedridden, incontinent, or requiring constant nursing care and attention), and a score of 6 indicating death. Death certificates were obtained for deceased participants. Hospital data were abstracted for all events, and the time, place and cause of death were recorded.

Overnight fasting blood samples were obtained. For CATIS participants, blood samples were obtained before intervention. Plasma (serum) and white blood cell samples were isolated, and DNA was extracted from leukocytes for all participants. The specimens were frozen at −80°C until laboratory testing. Serum lipids, plasma glucose, and other regular clinical laboratory measurements were performed on fresh blood samples at each participating center.

### DNA Methylation Sequencing

The current gold standard for the detection of DNA methylation is bisulfite sequencing. To measure the DNA methylation levels of the 20 genes, targeted bisulfite sequencing (MethylTarget™) developed by Genesky Biotechnologies, Inc., (Shanghai, China), was carried out as previously described (30). Briefly, CpG islands adjacent to the promoter regions (from 2 kilobases upstream of the transcription start site to 1 kilobase downstream of the first exon) of these genes were analyzed, and based on these CpG islands, 44 targets from CpG islands in 17 genes were sequenced (*ARHGAP24, SH3TC2* and *SLC22A7* were not tested because no CpG islands were found). The genomic information and primers can be found in Supplementary Tables 1, 2, respectively. Genomic DNA that passed quality control (concentration ≥20 ng/μL, total DNA ≥1 μg, OD260/280 = 1.7–2.0, OD260/230 ≥1.8) was bisulfite converted using the EZ DNA Methylation-Gold Kit (ZYMO, CA, USA), and the targeted DNA sequences were amplified by PCR. The products were sequenced on an Illumina MiSeq benchtop sequencer (Illumina, CA, United States). The methylation level at each methylation site was calculated as the percentage of methylated cytosines over all cytosines tested. The average methylation level for a target was calculated using the methylation levels of all measured methylation sites within the genomic fragment. The average methylation level for a gene was calculated using the methylation levels of all measured methylation sites within the gene. To test the reliability of methylation levels for each methylation site, 20 samples were randomly selected for duplicate detection.

### Statistical Analysis

The differences in baseline risk factors between cases and controls were compared using Student's *t*-test for continuous variables and χ^2^ tests for categorical variables. The normality of the methylation level data was judged using the Shapiro-Wilk normality test. Methylation level is not normally distributed. Therefore, the difference of the methylation levels between ischemic stroke cases and controls were assessed by Wilcoxon's two sample test. Methylation levels of each gene in ischemic stroke cases and controls were compared and bar plots depict the means, and the error bars in the figures represent SDs.

For ischemic stroke risk, logistic regression models were used to calculate the odds ratios (ORs) and 95% confidence intervals (CIs) per 5% increase in methylation levels in stage one, stage two and the combined samples. Potential covariates such as age, sex, current smoking, alcohol drinking, BMI, hypertension, type 2 diabetes, TC, TG, HDL-C and LDL-C were included in the multivariate models for case-control analysis. The analyses were performed in the combined sample and subgroups stratified by sex. Receiver operating characteristic (ROC) curves were plotted, and the areas under the curves (AUCs) were calculated to evaluate the diagnostic value of methylation levels. The optimal methylation level cutoff point was determined according to the ROC curve and Youden index analysis to classify the study participants into hypermethylated or hypomethylated groups for each target. Continuous net reclassification improvement (NRI) and integrated discrimination improvement (IDI) were calculated to evaluate the ability of methylations to reclassify risks based on a basic model with established risk factors (31).

For ischemic stroke prognosis, linear regression models were used to assess the associations between mRS score and methylation levels. Logistic regression models were used to calculate ORs and 95% CIs for major disability and death. Potential covariates such as age, sex, admission NIHSS score, SBP, eGFR, current smoking, alcohol consumption, use of antihypertensive medications, family history of stroke, ischemic stroke subtype, randomized treatment, and history of hypertension, hyperlipidemia, diabetes mellitus, and coronary heart disease were included in the multivariate models. A Cox proportional hazards model was used to determine the association between methylation levels and the risk of death within 3 months of onset adjusted for potential confounders. The survival time was from onset to death or last investigation (censored). Survival curves for patients in tertile categories of methylation levels were plotted and tested by using the Mantel-Haenszel test. All analyses were performed using the R language program (Version 3.5.0). We performed functional annotation clustering analysis to identify possible biological pathways for the associated genes by using the DAVID tools (https://david.ncifcrf.gov/home.jsp).

## Results

### Case-Control Study for Ischemic Stroke Risk

#### Association Between Methylation Levels and Ischemic Stroke

The basic characteristics of the study participants are presented in Table 1. A total of 271 ischemic stroke cases and 323 controls were included in stage 1, and 852 ischemic stroke cases and 925 controls were included in stage 2. Compared with the controls, mean BMI, SBP, DBP, TC, LDL-C, TG and FBG levels were all higher, while HDL-C levels were lower in ischemic stroke patients in both stage samples (Table 1). The associations between methylation levels and ischemic stroke were examined in two-stage case-control samples.

**Table 1:** Characteristics of the ischemic stroke cases and controls.

**Characteristics**	**Stage 1**			**Stage 2**		
	**Cases (*n* = 271)**	**Controls (*n* = 323)**	***P-*values**	**Cases (*n* = 852)**	**Controls (*n* = 925)**	***P-*values**
Age, year	57.3 ± 10.46	57.0 ± 10.83	0.7238	62.5 ± 12.09	62.2 ± 12.17	0.6632
Male, %	57.34	56.43	0.8683	53.10	54.26	0.6579
Smokers, %	40.11	40.35	1.000	34.47	37.54	0.1943
Drinkers, %	35.31	24.27	0.0019	26.03	27.29	0.5845
SBP, mmHg	173.2 ± 18.88	120.2 ± 13.56	9.0 × 10^−195^	171.9 ± 17.36	137.7 ± 23.20	3.9 × 10^−203^
DBP, mmHg	101.8 ± 11.29	72.6 ± 7.98	1.6 × 10^−178^	98.9 ± 10.96	81.7 ± 11.95	1.3 × 10^−174^
Hypertension, %	100	17.5	2.9 × 10^−72^	100	46.9	3.2 × 10^−173^
BMI, kg/m^2^	25.3 ± 3.02	21.7 ± 2.77	2.5 × 10^−49^	25.1 ± 3.39	22.4 ± 3.37	2.6 × 10^−60^
TC, mmol/L	5.14 ± 1.20	4.47 ± 0.87	6.0 × 10^−16^	5.12 ± 1.16	4.67 ± 0.96	1.3 × 10^−18^
TG, mmol/L	1.96 ± 1.29	1.33 ± 0.94	9.6 × 10^−13^	1.89 ± 4.96	1.56 ± 1.14	0.0448
HDL-C, mmol/L	1.28 ± 0.53	1.40 ± 0.31	0.0005	1.30 ± 0.41	1.34 ± 0.33	0.0278
LDL-C, mmol/L	3.01 ± 1.07	2.49 ± 0.71	3.7 × 10^−13^	2.94 ± 0.98	2.66 ± 0.78	8.2 × 10^−13^
FBG, mmol/L	6.89 ± 2.75	5.07 ± 0.64	7.8 × 10^−30^	6.78 ± 2.82	5.03 ± 1.17	4.6 × 10^−62^
Type 2 diabetes, %	17.0	0	5.0 × 10^−14^	19.4	2.9	1.6 × 10^−28^

*Compared with the controls, the mean BMI, SBP, DBP, TC, LDL-C, TG and GLU levels were all higher, while HDL-C level was lower in ischemic stroke patients in both stages*.

**Figure 1:**
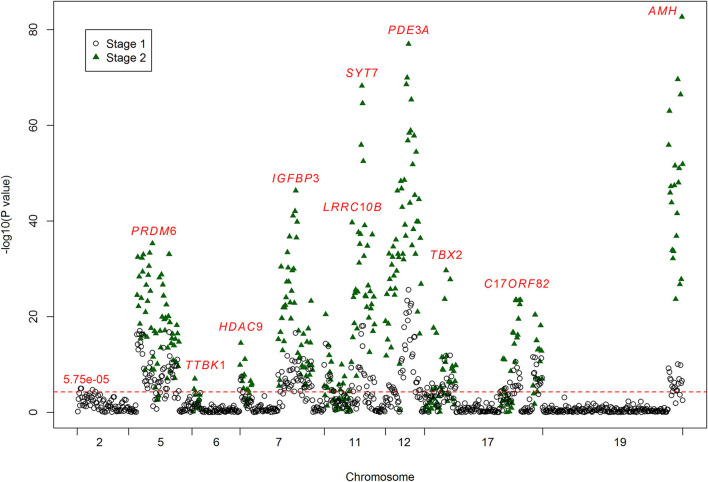
Results for the associations between DNA methylation and ischemic stroke. The crude *P*-values for the associations between DNA methylation and ischemic stroke. The x-axis represents the chromosome positions. The y-axis shows the –log_10_ *P*-values for the associations between the levels of DNA methylation sites and ischemic stroke. A total of 866 methylation sites were analyzed in stage one (black circles), and 224 of them passed the significance threshold of 5.75 × 10^−5^ (red line). A total of 377 methylation sites were analyzed in stage two (green triangles), and 296 of them passed the significance threshold of 5.75 × 10^−5^. The associated methylation sites tend to cluster in targets.

In the first stage, we tested 866 DNA methylation sites in 44 targets for the 17 genes (Supplementary Table 1). According to the duplicate detection in 20 individuals, there was no significant difference between the two tests for the methylation levels of each of these 866 sites. According to the Wilcoxon's two sample test, methylation levels of 224 sites were significantly associated with ischemic stroke (*P* < 5.75 × 10^−5^, Bonferroni correction) (Figure 1). The significant sites were located in 21 targets for 10 genes, including *AMH* (number of significant targets in the gene: 1), *C17orf82* (3), *HDAC9* (1), *IGFBP3* (2), *LRRC10B* (2), *PDE3A* (3), *PRDM6* (4), *SYT7* (1), *TBX2* (3) and *TTBK1* (1). Methylation levels of most of the sites in these targets were associated with ischemic stroke. Mean methylation levels of the targets were associated with ischemic stroke, and higher methylation levels of the targets were associated with a lower risk of ischemic stroke (Table 2). These 21 targets were subsequently sequenced in the samples of stage two.

**Table 2:** The associations between methylation levels of each target and ischemic stroke.

**Targets**	**CHR**	**Methylation target position**	**Length**	Number of sites^*^	**Stage 1**		**Stage 2**		**Combined**	
		**(Start—End, hg19)**	**(bp)**		**OR (95% CI)**	***P*-value**	**OR (95% CI)**	***P*-value**	**OR (95% CI)**	***P*-value**
PRDM6_2	5	122424419–122424583	165	12 (12)	0.83 (0.73, 0.95)	6.55 × 10^−03^	0.47 (0.41, 0.55)	1.02 × 10^−21^	0.57 (0.51, 0.64)	3.50 × 10^−20^
PRDM6_3	5	122424917–122425111	195	19 (18)	0.85 (0.75, 0.96)	1.04 × 10^−02^	0.50 (0.44, 0.57)	2.73 × 10^−24^	0.56 (0.50, 0.62)	1.57 × 10^−27^
PRDM6_4	5	122425618–122425772	155	11 (11)	0.61 (0.49, 0.76)	1.16 × 10^−05^	0.34 (0.26, 0.43)	1.60 × 10^−17^	0.36 (0.30, 0.43)	1.58 × 10^−25^
PRDM6_5	5	122426218–122426384	167	19 (19)	0.81 (0.74, 0.89)	2.19 × 10^−05^	0.73 (0.67, 0.80)	1.59 × 10^−11^	0.72 (0.67, 0.77)	3.54 × 10^−20^
TTBK1	6	43211012–43211204	193	12 (3)	0.97 (0.90, 1.04)	3.99 × 10^−01^	1.13 (1.06, 1.21)	5.56 × 10^−04^	1.07 (1.01, 1.14)	1.48 × 10^−02^
HDAC9_1	7	18125758–18126026	269	18 (14)	0.82 (0.73, 0.93)	2.12 × 10^−03^	0.60 (0.53, 0.66)	1.69 × 10^−20^	0.62 (0.57, 0.68)	2.12 × 10^−25^
IGFBP3_2	7	45960262–45960480	219	28 (28)	0.92 (0.82, 1.04)	1.79 × 10^−01^	0.64 (0.58, 0.70)	2.20 × 10^−20^	0.66 (0.61, 0.71)	1.04 × 10^−25^
IGFBP3_3	7	45960753–45960958	206	22 (22)	0.92 (0.83, 1.02)	1.12 × 10^−01^	0.64 (0.57, 0.71)	2.65 × 10^−15^	0.71 (0.65, 0.77)	2.71 × 10^−14^
LRRC10B_1	11	61275868–61276114	247	19 (13)	0.70 (0.55, 0.90)	5.73 × 10^−03^	0.49 (0.40, 0.60)	6.81 × 10^−12^	0.49 (0.41, 0.58)	2.95 × 10^−16^
LRRC10B_2	11	61276891–61277075	185	23 (11)	0.85 (0.74, 0.97)	1.85 × 10^−02^	0.69 (0.59, 0.80)	2.39 × 10^−06^	0.69 (0.61, 0.78)	1.36 × 10^−09^
SYT7_1	11	61348165–61348388	224	31 (31)	0.71 (0.88, 0.58)	1.20 × 10^−03^	0.22 (0.18, 0.28)	1.13 × 10^−38^	0.29 (0.25, 0.34)	2.32 × 10^−44^
PDE3A_1	12	20521690–20521926	237	22 (21)	0.94 (0.88, 1.00)	4.75 × 10^−02^	0.55 (0.50, 0.60)	2.57 × 10^−33^	0.68 (0.64, 0.73)	7.60 × 10^−28^
PDE3A_2	12	20522200–20522368	169	16 (16)	0.86 (0.80, 0.93)	1.26 × 10^−04^	0.58 (0.53, 0.63)	5.24 × 10^−37^	0.65 (0.61, 0.69)	1.16 × 10^−36^
PDE3A_3	12	20522826–20523046	221	14 (14)	0.86 (0.78, 0.95)	3.60 × 10^−03^	0.49 (0.44, 0.55)	1.82 × 10^−36^	0.56 (0.52, 0.61)	1.13 × 10^−38^
TBX2_1	17	59475132–59475331	200	13 (11)	0.99 (0.97, 1.01)	2.26 × 10^−01^	0.97 (0.94, 1.00)	2.06 × 10^−02^	0.98 (0.96, 1.00)	2.28 × 10^−02^
TBX2_2	17	59475409–59475636	228	11 (5)	0.91 (0.87, 0.95)	5.73 × 10^−05^	0.89 (0.84, 0.94)	1.40 × 10^−04^	0.90 (0.86, 0.94)	8.54 × 10^−06^
TBX2_3	17	59476394–59476620	227	22 (19)	0.84 (0.73, 0.96)	9.46 × 10^−03^	0.52 (0.45, 0.60)	1.03 × 10^−19^	0.57 (0.51, 0.64)	4.08 × 10^−22^
C17orf82_2	17	59488091–59488288	198	15 (12)	0.81 (0.67, 0.99)	4.10 × 10^−02^	0.95 (0.78, 1.15)	5.87 × 10^−01^	0.89 (0.76, 1.05)	1.68 × 10^−01^
C17orf82_3	17	59488839–59489012	174	15 (13)	0.81 (0.72, 0.91)	3.74 × 10^−04^	0.67 (0.59, 0.75)	3.70 × 10^−11^	0.67 (0.61, 0.74)	1.79 × 10^−16^
C17orf82_5	17	59490117–59490276	160	14 (14)	0.76 (0.61, 0.94)	1.20 × 10^−02^	0.46 (0.36, 0.60)	3.88 × 10^−09^	0.53 (0.44, 0.65)	2.82 × 10^−10^
AMH_3	19	2252326–2252568	243	21 (21)	0.93 (0.86, 1.00)	6.75 × 10^−02^	0.52 (0.47, 0.57)	4.05 × 10^−38^	0.63 (0.59, 0.68)	1.59 × 10^−33^

*CHR, Chromosome; CI, Confidence interval; OR, Odds ratio. ORs and 95% CIs per 5% increase in methylation levels were calculated. The associations were adjusted for age, sex, smoking, drinking, BMI, hypertension, type 2 diabetes, TC, TG, HDL-C and LDL-C*.

* *Number of methylation sites tested in each target. Numbers in the bracket indicate the number of methylation sites that were significantly associated with ischemic stroke in each of the targets. The methylation level of a target was the mean methylation level of the sites in the target*.

In the second stage of the study, 377 methylation sites were analyzed, and 296 of them passed the significance threshold of 5.75 × 10^−5^ (Figure 1). Indeed, 299 sites could be considered significant (*P* < 1.33 × 10^−4^, Bonferroni correction). The significant ischemic stroke-associated sites were located in each of the 21 targets. Almost all of the methylation sites that were significantly associated with ischemic stroke in stage one were significant in stage two, except for 4 sites in *TBX2* and 1 site in *TTBK1*. Additionally, 89 sites that did not pass the significance threshold in stage one were significant in stage two. At target level, 17 targets were significantly associated with ischemic stroke (Table 2). The associated methylations seemed to be hypomethylated in ischemic stroke cases. TTBK1, TBX2_1, TBX2_2 and C17orf82_2 were not significant. The effect of *TTBK1* methylation on ischemic stroke was inconsistent between samples from stage one and stage two. According to the results from stage one and stage two, we confirmed that higher methylation levels of 17 targets in *AMH, C17orf82, HDAC9, IGFBP3, LRRC10B, PDE3A, PRDM6, SYT7* and *TBX2* genes were associated with a lower risk of ischemic stroke.

Furthermore, the effects of methylation on ischemic stroke risk were evaluated in the combined sample of 1,123 cases and 1,257 controls (Table 2). The results showed that the mean methylation levels of *AMH, C17orf82, HDAC9, IGFBP3, LRRC10B, PDE3A, PRDM6, SYT7* and *TBX2* were significantly (Wilcoxon's two sample test) lower in ischemic stroke cases than in controls (Supplementary Figure 1). For most of the 21 targets, methylation levels of most (more than 60%) sites tested in each target were associated with ischemic stroke, except for TTBK1 (25%), LRRC10B_2 (48%) and TBX2_2 (46%) (Table 2). Indeed, the associations between the methylation levels of TTBK1, TBX2_1 and C17orf82_2 and ischemic stroke were not significant at target level. Higher methylation levels of 18 targets in *AMH, C17orf82, HDAC9, IGFBP3, LRRC10B, PDE3A, PRDM6, SYT7* and *TBX2* genes were associated with a lower risk of ischemic stroke. Each 5% higher methylation level of targets PRDM6_4, LRRC10B_1 and SYT7_1 was associated with a 64% (adjusted OR = 0.36, 95% CI: 0.30–0.43), 51% (adjusted OR = 0.49, 95% CI: 0.41–0.58) and 71% (adjusted OR = 0.29, 95% CI: 0.25–0.34) decreased risk for ischemic stroke, respectively (Table 2). The associations were mostly consistent between male (656 cases and 696 controls) and female (551 cases and 573 controls) participants (Supplementary Table 3). We also obtained similar results for most of the targets when we restricted the control sample to hypertensive or normotensive participants only, except for LRRC10B_2 (Supplementary Figure 2).

#### Multiple Hypomethylated Genes and Ischemic Stroke

**Figure 2:**
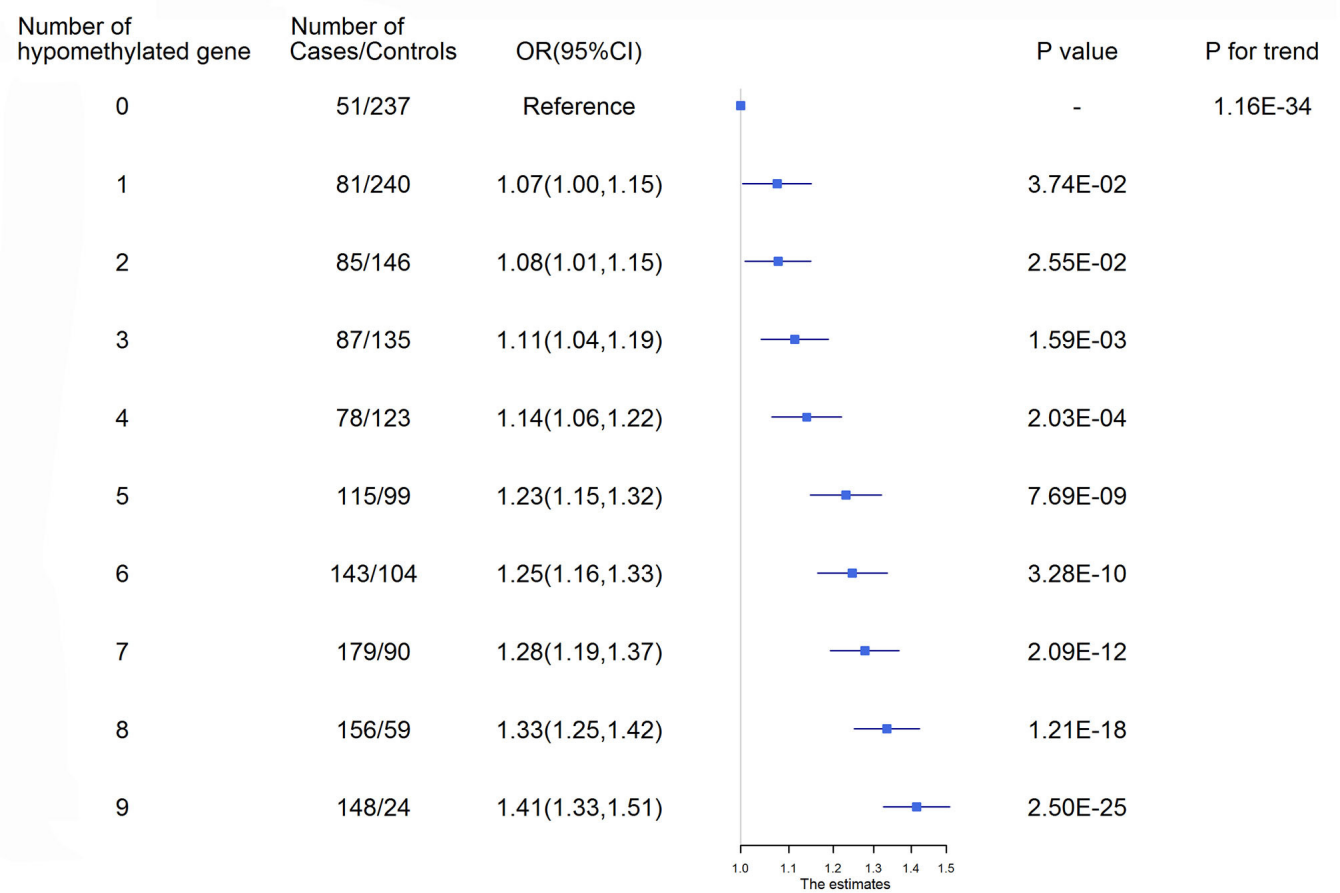
Ischemic stroke risk according to the number of hypomethylated genes. ROC curves were used to obtain the optimal cutoff points for the representative targets of 9 genes, including AMH_3 (<3.21%), C17orf82_3 (<3.35%), HDAC9_1 (<1.88%), IGFBP3_2 (<3.90%), LRRC10B_1 (<1.09%), PDE3A_3 (<2.47%), PRDM6_3 (<2.01%), SYT7_1 (<1.98%) and TBX2_3 (<3.00%), to define hypomethylation. CI, Confidence interval; OR, Odds ratio.

ROC curves were used to obtain the optimal cutoff points for the representative targets of 9 genes (*AMH, C17orf82, HDAC9, IGFBP3, LRRC10B, PDE3A, PRDM6, SYT7* and *TBX2*) to define hypomethylation. Participants with a larger number of hypomethylated targets had an increased risk of ischemic stroke (*P* for trend = 1.16 × 10^−34^, Figure 2). A total of 172 (6.95%) participants had all 9 hypomethylated genes and had the highest risk. Compared to the participants without any hypomethylated genes, the OR (95% CI) of those with 9 hypomethylated targets was 1.41 (1.33–1.51) for ischemic stroke after adjustment for covariates.

#### Ischemic Stroke Risk Prediction

The AUCs calculated by *AMH* (methylation level of AMH_3), *PDE3A* (mean level of PDE3A_1, PDE3A_2 and PDE3A_3), *PRDM6* (mean level of PRDM6_2, PRDM6_3, PRDM6_4 and PRDM6_5), and *SYT7* (level of SYT7_1) gene methylation levels were larger than 0.7 (Supplementary Figure 3). The predictive ability for ischemic stroke risk by the joint use of methylation levels, BMI, SBP, DBP, GLU and TC was significantly increased compared to the joint use of BMI, SBP, DBP, GLU and TC (*P* = 2.25 × 10^−10^). In addition, adding methylation levels of each of the 18 targets for the 9 genes (i.e., *AMH, C17orf82, HDAC9, IGFBP3, LRRC10B, PDE3A, PRDM6, SYT7* and *TBX2*) to the basic model of traditional risk factors significantly improved the risk stratification for ischemic stroke, as shown by increases in continuous NRIs ranging from 16.00% (for TBX2_2) to 59.91% (for SYT7_1) and IDIs ranging from 0.43% (for TBX2_2) to 4.09% (for SYT7_1) (Supplementary Table 4).

### Follow-Up Study for Ischemic Stroke Prognosis

#### Association Between Methylation Levels and mRS Score

The baseline characteristics of the 1,123 ischemic stroke patients are presented in Table 3. The patients were all hypertensives. The mean age was 61 and 54.3% were males. Among these study participants, a total of 339 had major disability (mRS score 3–5) at 3 months after onset, and 44 patients died within 3 months of onset.

**Table 3:** Baseline characteristics of ischemic stroke patients.

	**Total (*n* = 1,123)**
**Demographics**	
Age, year	61.0 ± 11.9
Male sex	656 (54.3%)
Current cigarette smoking	436 (36.1%)
Current alcohol drinking	347 (28.7%)
**Clinical features**	
Baseline systolic blood pressure, mm Hg	168.4 ± 17.1
Baseline diastolic blood pressure, mm Hg	97.8 ± 10.9
Estimated glomerular filtration rate, mL/min/1.73 m^2^	105.0 ± 117.1
Baseline NIH Stroke Scale score	3.1 ± 1.1
**Medical history**	
History of hypertension	953 (79.0%)
History of hyperlipidemia	86 (7.1%)
History of diabetes mellitus	227 (18.8%)
History of coronary heart disease	118 (9.8%)
Family history of stroke	268 (22.2%)
Prior use of antihypertensive drugs	470 (38.9%)
**Ischemic stroke subtype**	
Thrombotic	1,005 (83.3%)
Embolic	54 (4.5%)
Lacunar	113 (9.4%)

*The 1,123 ischemic stroke cases were all hypertensive, and their SBP levels were all higher than 140 mmHg, while 184 cases had DBP levels <90 mmHg. The mean age was 61 and 54.3% were males. Among these study participants, a total of 339 had major disability (mRS score 3–5) at 3 months after onset, and 44 patients died within 3 months of onset*.

After adjusting for covariates, the methylation levels of 5 sites in *PRDM6*, 1 site in *IGFBP3*, 2 sites in *PDE3A* and 1 site in *AMH* passed the significance threshold of 1.33 × 10^−4^ (Supplementary Figure 4). For the 21 targets, associations between methylation levels of AMH_3, HDAC9_1, IGFBP3_3, PDE3A_2, PRDM6_2, PRDM6_3 and mRS score were significant after adjustment for covariates (*P* <2.38 × 10^−3^, Bonferroni correction), and nominal evidence was found for the associations between other 8 targets and mRS score (Supplementary Table 5). Methylation levels of these targets and mRS scores were negatively correlated.

#### Association Between Methylation Levels and Major Disability and Death

The methylation level of only one site in *PDE3A* was significantly associated with major disability. Methylation levels of one site in *C17orf82* and 2 sites in *TBX2* were significantly associated with death (Supplementary Figure 4). For the 21 targets, methylation levels of PDE3A_2 were significantly associated with major disability and death (mRS score ≥3) after adjusting for covariates (*P* < 2.38 × 10^−3^). Methylation levels of targets AMH_3, C17orf82_5, PRDM6_2 and TBX2_3 were significantly associated with death (Supplementary Table 5).

**Figure 3:**
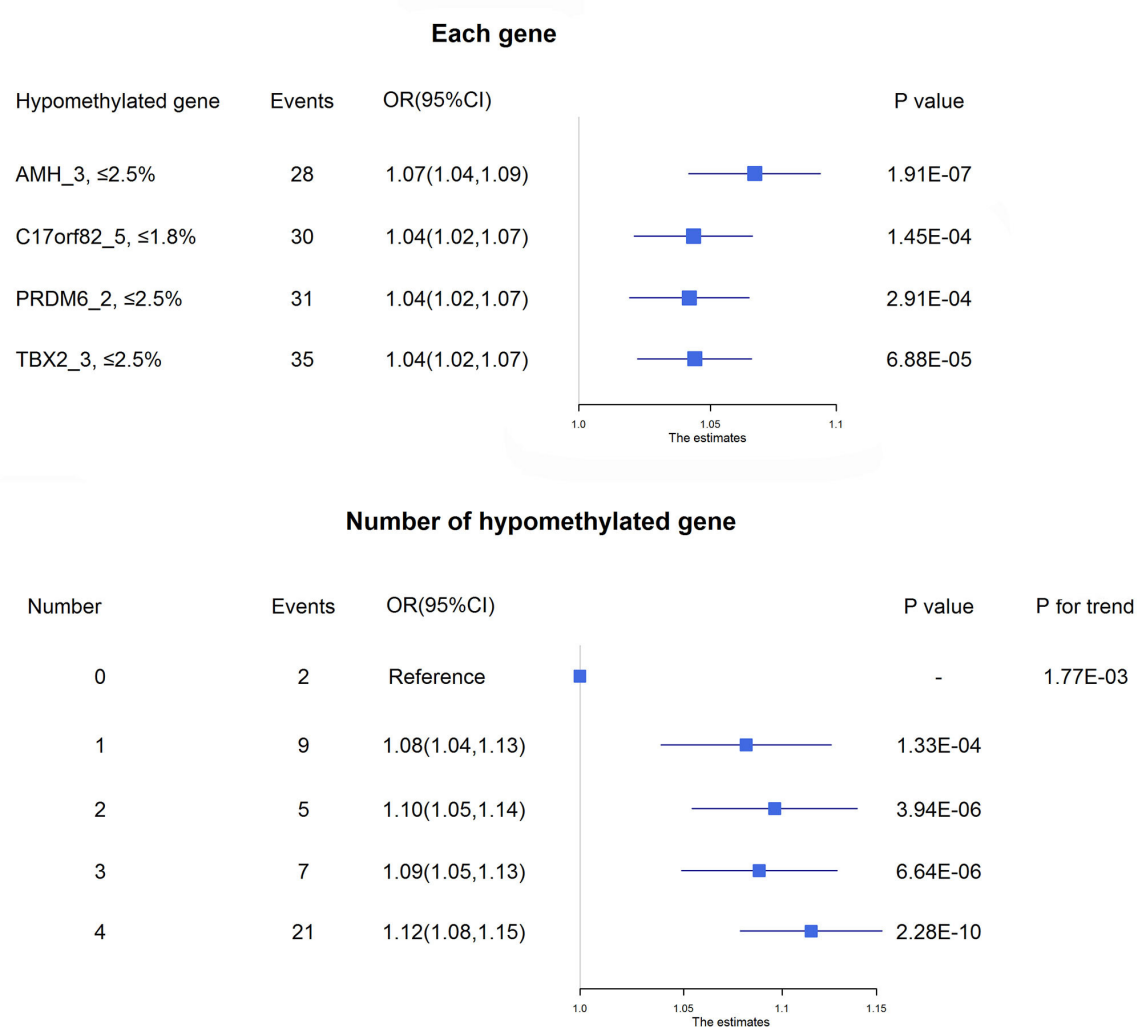
Association between DNA methylation and 3-month mortality. The upper panel shows the adjusted ORs and corresponding 95% CIs for the associations between hypomethylation of each target and 3-month mortality. The lower panel shows the adjusted ORs and corresponding 95% CIs for 3-month mortality according to the number of hypomethylated targets among acute ischemic stroke patients.

Four targets seemed to be hypomethylated in ischemic stroke patients who experienced death within 3 months of onset. ROC curves were used to obtain the optimal cutoff points for these 4 targets, including AMH_3 (methylation level ≤2.5%), C17orf82_5 (≤1.8%), PRDM6_2 (≤2.5%) and TBX2_3 (≤2.5%), to define hypomethylation. Compared with patients without hypomethylation of corresponding targets, hypomethylation of AMH_3 was associated with a 7% increased risk of death, and hypomethylation of C17orf82_5, PRDM6_2 and TBX2_3 was associated with a 4% increased risk of death (Figure 3). Patients with a higher number of hypomethylated targets had a higher risk (*P* for trend = 1.77 × 10^−3^). A total of 152 (12.6%) participants had all 4 hypomethylated targets, and 21 (13.8%) events were observed. Compared with patients without any hypomethylated targets, the OR (95% CI) for those with 4 hypomethylated targets was 1.12 (1.08–1.15) after adjustment for covariates (*P* = 2.28 × 10^−10^, Figure 3).

The prediction ability for death within 3 months of onset by the joint use of methylation levels of AMH_3, C17orf82_5, PRDM6_2 and TBX2_3, admission NIHSS score and age was significantly (*P* = 0.0124) increased compared to the joint use of admission NIHSS score and age, with AUCs (95% CIs) of 0.84 (0.77, 0.91) and 0.79 (0.70, 0.87), respectively (Supplementary Figure 5). Adding the methylation levels of targets of AMH_3, C17orf82_5, PRDM6_2 and TBX2_3 to the basic model of traditional risk factors substantially improved the risk stratification for ischemic stroke, as shown by increases in continuous NRIs and IDIs. Simultaneously adding these 4 targets to the model increased 71.57% (95% CI: 43.25–99.89%) in continuous NRIs and 6.36% (3.19–9.52%) in IDIs (Supplementary Table 6).

#### Association Between Methylation Levels and Survival

**Figure 4:**
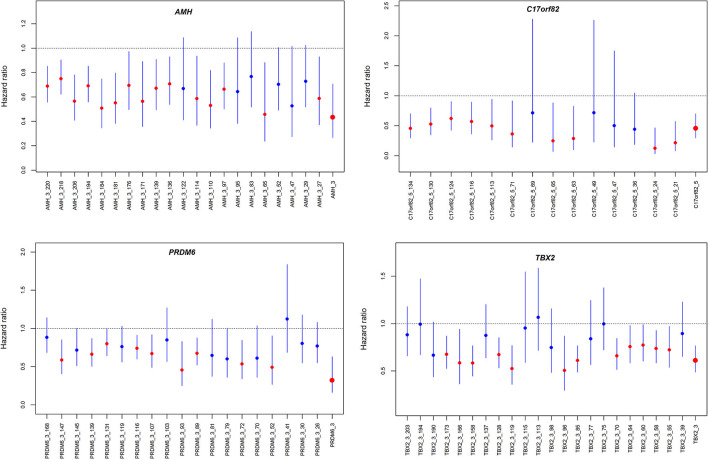
Hazard ratios for the association between DNA methylation and 3-month mortality. The four panels show the hazard ratios for each of the CpG sites in the four genes assessed by Cox proportional hazard models. The associations were adjusted for covariates.

**Figure 5:**
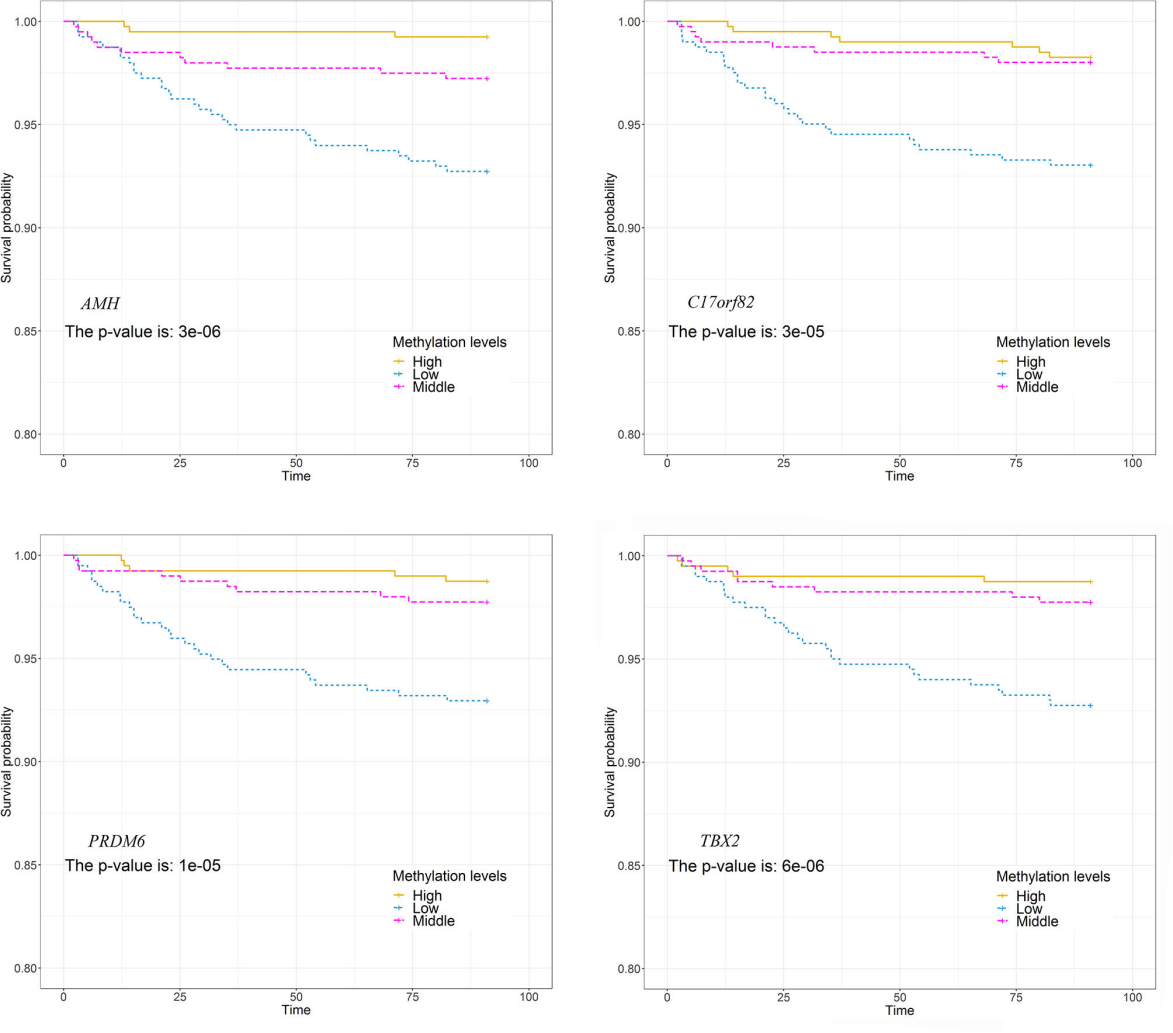
Association between DNA methylations and survival. The patients were separated into three groups by tertiles of methylation levels of each of the four targets. The survival rates between the three groups were compared. Patients who have higher methylation levels of these genes have higher survival rates than patients with lower methylation levels.

We observed significant associations between AMH_3, C17orf82_5, PRDM6_3 and TBX2_3 methylation levels and death within 3 months of onset in the Cox proportional hazards model after adjusting for covariates (Supplementary Table 5). For these four targets, 15 of the 21 (71.4%) sites in AMH_3, 10 of the 14 (71.4%) sites in C17orf82_5, 9 of the 19 (47.4%) sites in PRDM6_3 and 12 of the 22 (54.5%) sites in TBX2_3 were associated with 3-month mortality (Figure 4). Higher methylation levels were associated with a higher survival rate. Patients with acute ischemic stroke who have higher methylation levels of AMH_3, C17orf82_5, PRDM6_3 and TBX2_3 have higher survival rates than patients with lower methylation levels (Figure 5).

#### Interactions Between Methylations and Disease Severity on Mortality

**Figure 6:**
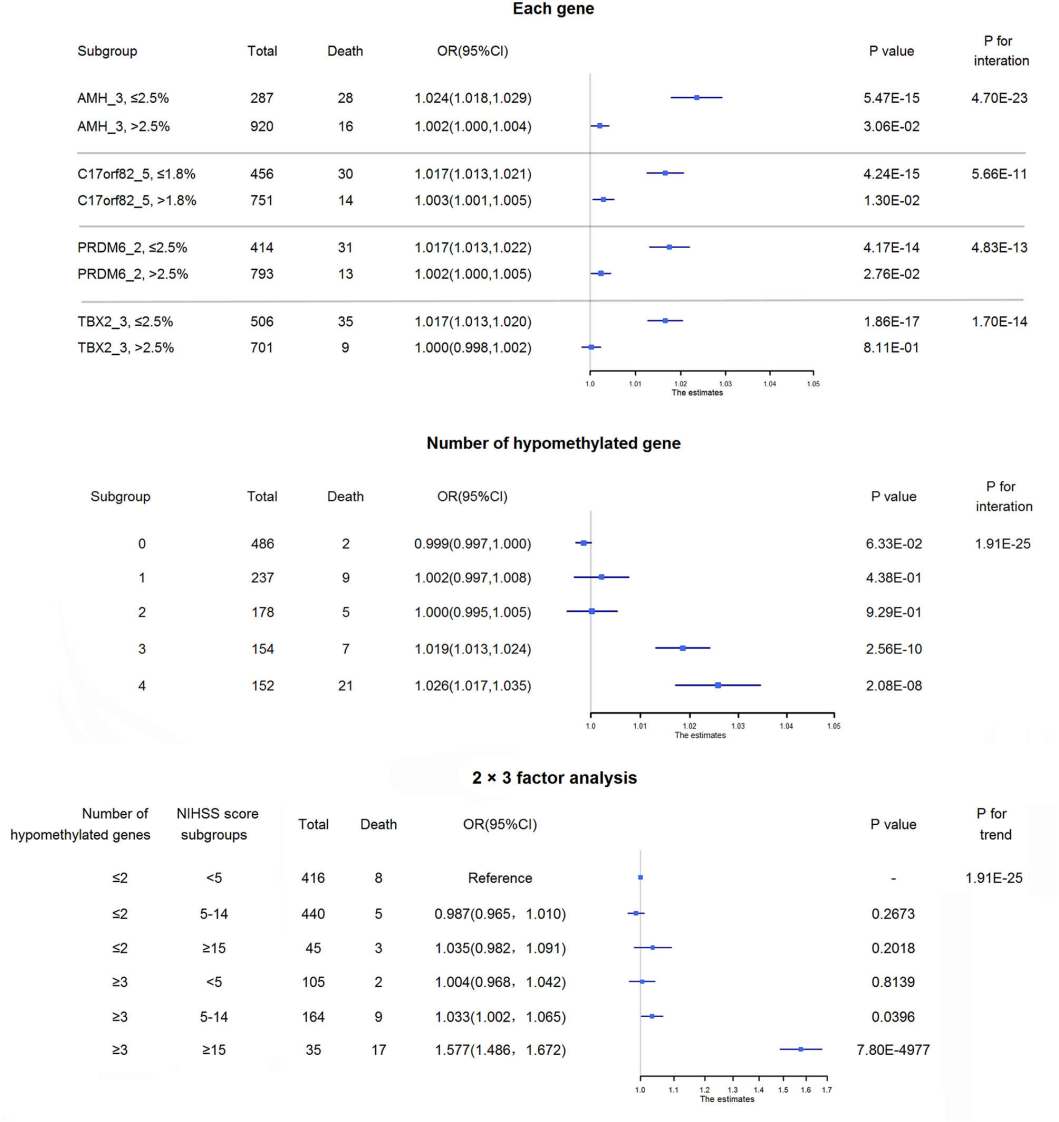
Interactions between methylations and disease severity on mortality. The upper panel shows the interaction effects between hypomethylation of each of the four targets and NIHSS score on 3-month mortality. The increased risk of death per 1-point increase in NIHSS score was higher in patients with hypomethylation of each of these 4 targets than in those without hypomethylation of the corresponding target. The middle panel shows the adjusted ORs and corresponding 95% CIs for the associations between NIHSS score and 3-month mortality among subgroups of acute ischemic stroke patients according to the number of hypomethylated targets. The increased risks of death per 1-point increase in NIHSS score were higher in patients with 3 or 4 hypomethylated targets than in those without hypomethylated targets. The lower panel shows the combined effect of the number of hypomethylated targets and disease severity on 3-month mortality. Patients were separated by the number of hypomethylated targets (separated by≤2 hypomethylated targets and ≥3 hypomethylated targets) and disease severity (minor stroke: NIHSS score <5, moderate stroke: NIHSS score 5–14, severe stroke: NIHSS score ≥15). Approximately 25.4% of ischemic stroke patients may have 3 or 4 hypomethylated genes, and nearly 10% of them would die within 3 months of onset, while the 3-month mortality for the overall patients was ~3%. Notably, for severe ischemic stroke patients with 3 or 4 hypomethylated genes, half of them died within 3 months of onset.

We observed significant interaction effects between hypomethylation of the four targets and NIHSS score on 3-month mortality (upper panel of Figure 6). We also observed a significant interaction effect between the number of hypomethylated targets and NIHSS score on the risk of death (middle panel of Figure 6). There were 154 (13.7%) and 152 (13.5%) ischemic stroke patients with 3 and 4 hypomethylated targets, and ~4.5 and 13.8% of them died within 3 months of onset, respectively. The increased risks of death per 1-point increase in NIHSS score were higher in patients with 3 or 4 hypomethylated targets.

We then conducted a 2 × 3 factor analysis to evaluate the combined effect of the number of hypomethylated targets and disease severity on mortality (lower panel of Figure 6). There were 35 (3.1%) severe ischemic stroke patients with ≥3 hypomethylated targets, and 48.6% of them died within 3 months of onset. The mortality of severe ischemic stroke patients with≤2 hypomethylated targets was only ~6.7% (3/45). Compared with the reference group (minor ischemic stroke patients with≤2 hypomethylated targets), severe ischemic stroke patients with ≥3 hypomethylated targets had an OR (95% CI) of 1.58 (1.49–1.67; *P* = 7.80 × 10^−4977^) for 3-month mortality.

#### Functional Clustering of the Identified Genes

In the functional annotation clustering analysis, we found that ischemic stroke risk- and prognosis-associated genes (*AMH, C17orf82, HDAC9, IGFBP3, PDE3A, PRDM6* and *TBX2*) were enriched in specific GO terms of biological process (Supplementary Table 7), including regulation of developmental process, muscle cell differentiation, muscle structure development, cell differentiation, cellular developmental process and regulation of muscle cell differentiation. *IGFBP3, PRDM6* and *HDAC9* are involved in all of these processes. *PDE3A* and *AMH* are involved in the regulation of developmental process, cell differentiation and cellular developmental process. *TBX2* is involved in the regulation of developmental process, muscle cell differentiation, muscle structure development, cell differentiation and cellular developmental process.

## Discussion

This study represents an effort to identify potential biomarkers for ischemic stroke among the Chinese Han population. We found hundreds of DNA methylation sites that were significantly associated with ischemic stroke in the stage one sample, and the associations were successfully validated in a larger stage two sample. This study also identified epigenetic factors for poor prognosis in a prospective study of acute ischemic stroke patients. This was the first study to report that hypomethylated genes inside the BP-associated loci identified by GWAS were associated with ischemic stroke risk and prognosis.

Blood can be utilized as a surrogate tissue for more pathologically relevant tissues involved in the study of cardiovascular disease (32). Studies have shown that blood DNA methylation plays important roles in stroke (33, 34). Hypertension is one of the most important risk factors for ischemic stroke; in addition, ischemic stroke shares many genetic and metabolic as well as environmental risk factors with hypertension. Among the 17 genes that were suggested to be related to BP levels by GWAS, DNA methylation levels of *AMH, C17orf82, HDAC9, IGFBP3, LRRC10B, PDE3A, PRDM6, SYT7* and *TBX2* in leukocytes were associated with risk of ischemic stroke among Chinese individuals in our study. Therefore, our study identified leukocyte DNA methylation as a newly epigenetic factor for ischemic stroke.

Some of the identified genes have been shown to be associated with stroke, e.g., the well-known stroke-related genes *HDAC9* and *IGFBP3*. *HDAC9* encodes histone deacetylase 9. The association between genetic variants of *HDAC9* and stroke has been confirmed by GWASs (5, 35). *HDAC9* is a gene involved in the signaling by NOTCH pathway and has been newly identified as an important determinant of the vascular smooth muscle cell phenotype and atherosclerotic aortic calcification (36). *HDAC9* genetic variants mediate their effects through increased *HDAC9* expression and increased ischemic stroke risk by promoting carotid atherosclerosis (37, 38). These studies have also shown that *HDAC9* expression is upregulated in carotid and femoral plaques (37) and is upregulated in the peripheral blood mononuclear cells of carriers of rs2107595, an ischemic stroke-risk allele (38). This result suggested that the overexpression of *HDAC9* increased ischemic stroke risk. As it is known that hypermethylation always suppresses gene expression, the findings of hypomethylated *HDAC9* in ischemic stroke patients in our study were consistent with previous findings from studies on gene expression. *IGFBP3* is a well-studied gene in the development IGF-1 receptor signaling pathway and the glucose/energy metabolism pathway. IGFBP3 modulates the actions of insulin-like growth factors, circulating hormones that influence vascular smooth muscle cell function. Circulating IGFBP3 (insulin-like growth factor binding protein 3) levels are positively associated with BP, stroke, carotid atherosclerosis and risk factors for several cardiovascular diseases (39–43).

Aside from these two known stroke genes, some of the remaining genes also play roles in the cardiovascular system. AMH (anti-Müllerian hormone) is a gonadal hormone present in the blood of men and premenopausal women. AMH has been shown to be a putative regulator of the cardiovascular system (44). DNA methylation in the *AMH* gene has been reported to have a strong effect on BP (6). Genetic variants in the *C17orf82* gene region were associated with serum urate concentrations (45) and chronic kidney disease (46). PDE3A (phosphodiesterase 3A) is a gene of the nitric oxide stimulates guanylate cyclase pathway and activation of cAMP-dependent PKA pathway and has long been known to be related to cardiovascular function by regulating vascular smooth muscle contraction and relaxation (47). Notably, a recent study has shown that *PDE3A* mutations cause Mendelian hypertension and brachydactyly type E (48).

Although hypertension increases the risk of ischemic stroke, BP levels were not associated with stroke severity and outcome, and BP reduction with antihypertensive medications did not reduce the likelihood of death or major disability among patients with acute ischemic stroke (28). However, some hypertension risk factors, e.g., high sensitivity C-reactive protein and matrix metalloproteinase-9, were found to be associated with poor outcome in ischemic stroke patients in our previous study (49), suggesting that factors associated with BP may have an impact on ischemic stroke prognosis. DNA methylation has been previously shown to be a potential biomarker of poor prognosis of stroke (22–25). However, whether DNA methylation of GWAS-identified BP-related genes is associated with clinical outcomes in ischemic stroke patients has not yet been examined. The associations between *AMH, C17orf82, PRDM6* and *TBX2* methylation levels and 3-month mortality after acute ischemic stroke were identified in this study. Therefore, the findings of this study showed that DNA methylation of some BP-related genes in leukocytes may be associated with clinical outcome after ischemic stroke.

We also found that severe ischemic stroke patients with more hypomethylated genes had higher mortality, while the 3-month mortality for severe ischemic stroke patients with≤2 hypomethylated targets was much lower. Therefore, by testing the methylation levels of the four genes, we were able to screen high-risk patients. We noticed that the proportion of severe ischemic stroke patients (*N* = 80) with 3 or 4 hypomethylated genes (*N* = 35) was high (~43.75%). Therefore, testing among severe ischemic stroke patients may be of clinical significance.

We noticed that most of the newly identified CpG sites overlapped with promoters or enhancers, the so-called GeneHancer regulatory elements (50). Moreover, the genomic regions of the CpG sites have long-range interactions with nearby promoters or enhancers (Supplementary Figure 6). For example, the significant methylation sites in target PRDM6_2 are located in a CpG island (chr5:122424338-122424539, CpG count: 21). This CpG island overlaps a promoter (chr5:122424113-122425192) at the 5′ region of *PRDM6*. The genomic region of this promoter interacts with two nearby enhancers (chr5:122433360-122436028 and chr5:122454855-122455750). It is known that DNA methylation in long-range interactive promoters and enhancers can affect long-range interactions and may be associated with the risk of disease (51, 52). The protein (a putative histone-lysine N-methyltransferase) encoded by *PRDM6* is involved in the regulation of contractile proteins in vascular smooth muscle cells (7). We found that *PRDM6* was differentially expressed between ischemic stroke cases and controls by analyzing data in the GEO database (Supplementary Figure 7) (53). The expression levels of *PRDM6* were higher in ischemic stroke patients than in controls. Based on this information, it seems that the identified DNA methylation may have the potential to regulate gene expression, and higher methylation levels of the *PRDM6* gene may lead to lower expression levels of *PRDM6* and contribute to a lower risk of ischemic stroke. Therefore, these methylations probably play causal roles in ischemic stroke.

Some limitations of this study should be mentioned. First, although we obtained significant findings in this study, we sequenced only a small fragment of the genomic region of the genes. To comprehensively evaluate the effects of methylation on stroke, whole genomic regions of the CpG islands are suggested to be sequenced in large samples. Second, the patients were from the CATIS trial in which patients with SBP between 140 mmHg and <220 mmHg were recruited. Therefore, selection bias might exist in this study. Third, although the identified methylations are located in promoters or enhancers, we did not perform a functional study to determine the effect of methylation on gene expression. We also have not validated the long-range interactions. Therefore, whether the methylation could functionally affect these long-range interactions and gene expression is uncertain.

## Conclusions

In conclusion, the present study showed that leukocyte DNA methylation of the *AMH, C17orf82, HDAC9, IGFBP3, LRRC10B, PDE3A, PRDM6, SYT7* and *TBX2* genes was significantly associated with the risk of ischemic stroke and identified prospective associations between lower methylation levels of the *PDE3A, AMH, C17orf82, PRDM6* and *TBX2* genes and poor outcomes within 3 months of the onset of ischemic stroke. This study increased our understanding of the role of DNA methylation in the pathogenesis of ischemic stroke and might propose new biomarkers. Given the reversible nature of epigenetic mechanisms, the identification of novel DNA methylations for ischemic stroke prognosis may provide promising avenues for preventions and therapeutics. Although we detected significant associations, further functional studies are needed to elucidate the mechanisms.

## Data Availability Statement

The datasets presented in this study can be found in the Supplementary Material.

## Ethics Statement

The studies involving human participants were reviewed and approved by Soochow University. The patients/participants provided their written informed consent to participate in this study.

## Author Contributions

HZ, XM, and YZ obtained funding. XM and YZ designed the study. HZ and XM performed the analyses and rafted the initial manuscript. AW, HP, DG, CZ, ZZ, and TX were involved in the study design and/or data collection. XM, HZ, and YZ have primary responsibility for the final content. YZ is the guarantor of this study. All authors contributed to the article and approved the submitted version.

## Funding

This study was supported by the Natural Science Foundation of China (82073636, 81773508, and 81673263), the Key Research Project (Social Development Plan) of Jiangsu Province (BE2016667), the Startup Fund from Soochow University (Q413900313 and Q413900412), and a Project of the Priority Academic Program Development of Jiangsu Higher Education Institutions.

## Conflict of Interest

The authors declare that the research was conducted in the absence of any commercial or financial relationships that could be construed as a potential conflict of interest.

## Publisher's Note

All claims expressed in this article are solely those of the authors and do not necessarily represent those of their affiliated organizations, or those of the publisher, the editors and the reviewers. Any product that may be evaluated in this article, or claim that may be made by its manufacturer, is not guaranteed or endorsed by the publisher.
