# Supplementary material for: Association of DNA Methylation in Blood Pressure-Related Genes With Ischemic Stroke Risk and Prognosis
Source: Front Cardiovasc Med. 2022 Mar 8;9:796245. doi: 10.3389/fcvm.2022.796245 (PMC8957103; doi:10.3389/fcvm.2022.796245)
Supplement: Supplementary file 2 [file Data_Sheet_2.docx]

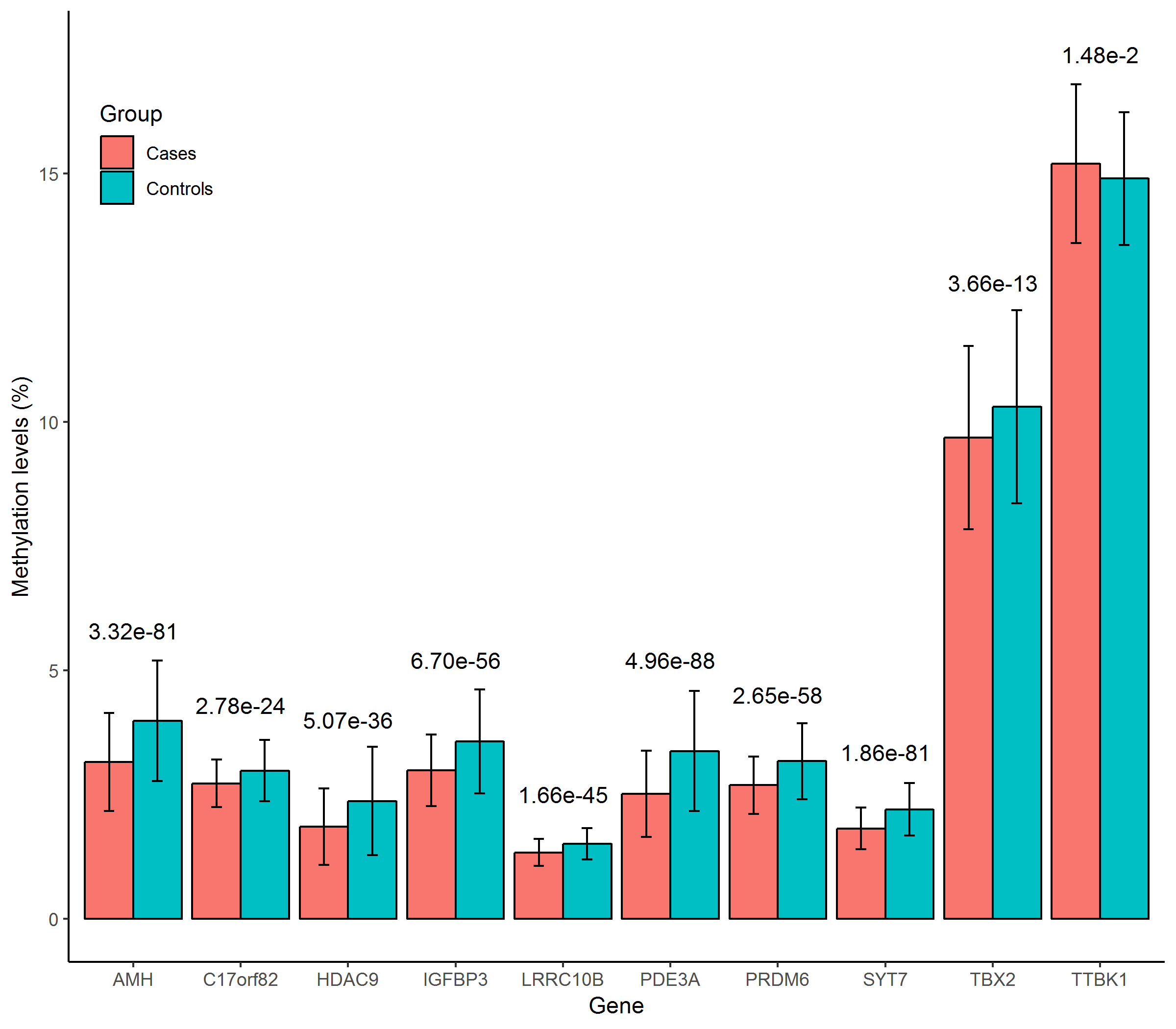


**Supplementary figure S1 Association between DNA methylations of each gene and ischemic stroke**

The difference of methylation levels for each gene between ischemic stroke cases and controls. The mean methylation levels for each gene were calculated using methylation levels of all measured methylation sites within the gene for each individual. The bar plots depict the means in the 1,207 cases and 1,269 controls samples, and the error bars in the figures represent standard deviations. These genes were significantly hypomethylated in ischemic stroke patients, except *TTBK1*. *P* values presented above the bars were assessed by Wilcoxon's two sample test.


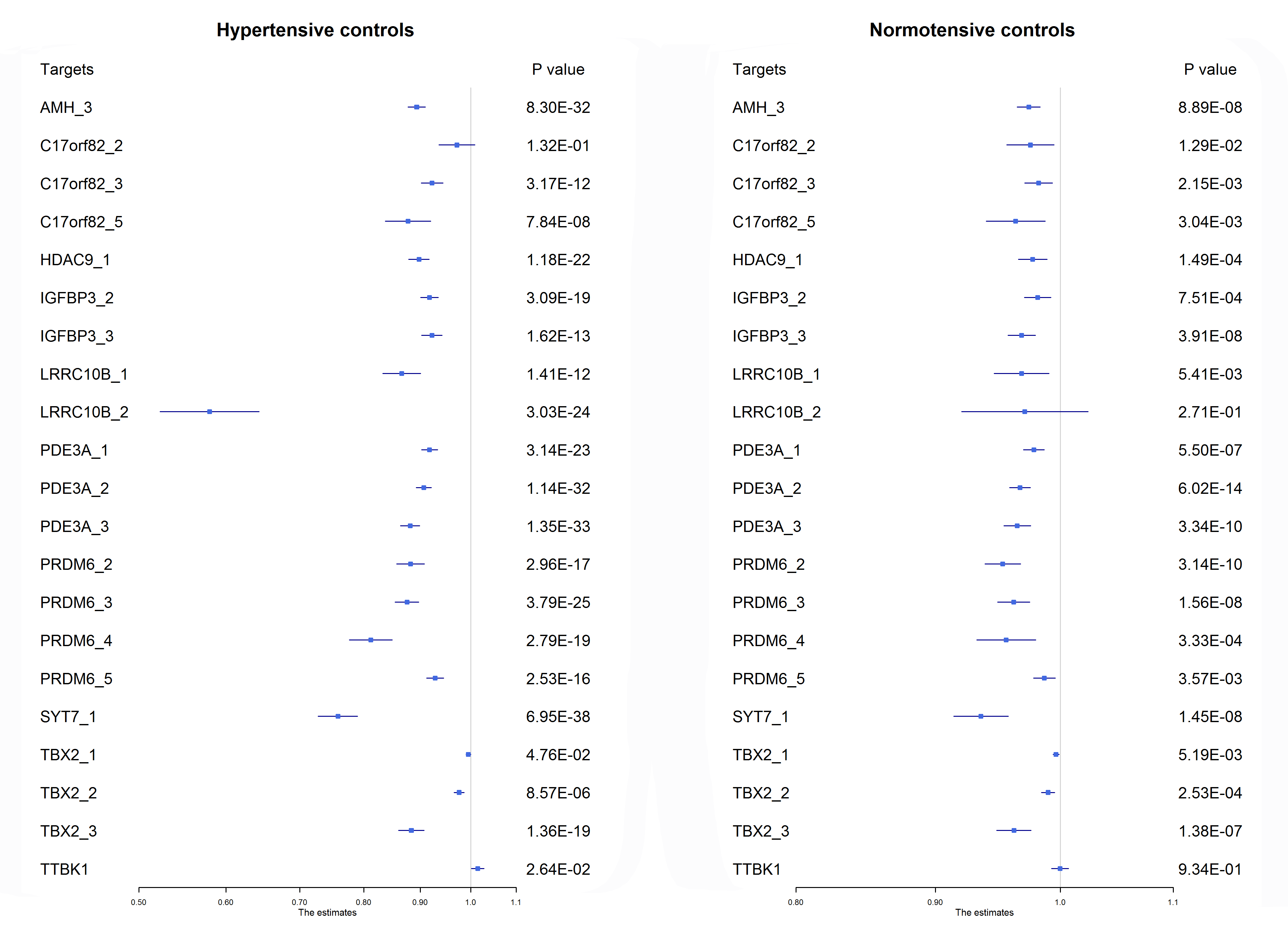


**Supplementary figure S2 Association between DNA methylations of each target and ischemic stroke**

The forest plots show the associations examined among ischemic stroke cases and hypertensive controls (left, n = 507) and the associations examined among ischemic stroke cases and normotensive controls (right, n = 762).


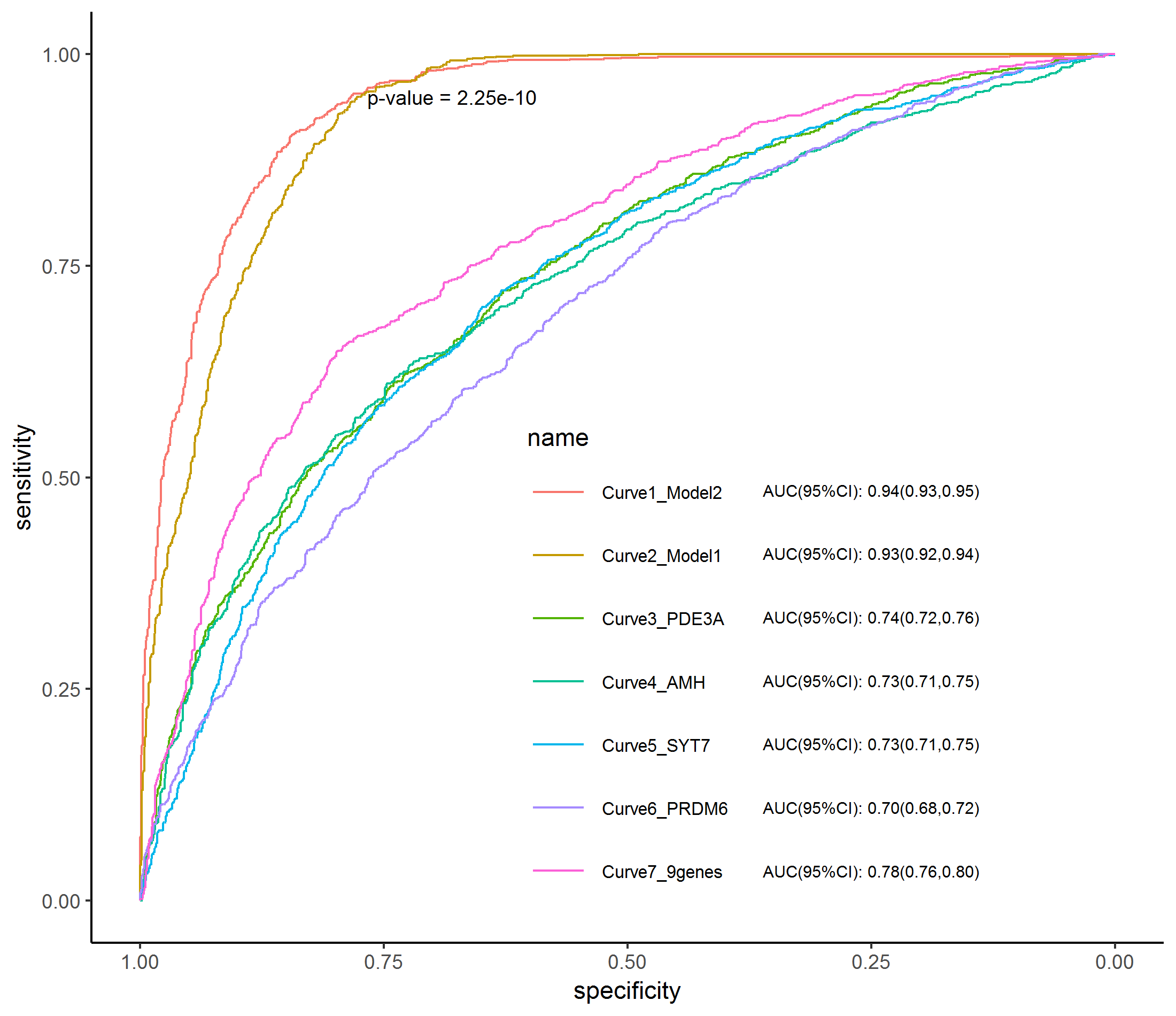


**Supplementary figure S3 ROC for ischemic stroke risk prediction in Chinese individuals**

Model 1 predicted ischemic stroke using BMI, SBP, DBP, GLU and TC. Model 2 predicted ischemic stroke by jointly using the methylation levels of the four genes, BMI, SBP, DBP, GLU and TC. DNA methylation levels significantly increased the prediction ability (*P* = 2.25 × 10^-10^). Curves 3, 4, 5 and 6 predicted ischemic stroke using only the *PDE3A*, *AMH*, *SYT7* and *PRDM6* methylation levels, respectively. Curves 7 predicted ischemic stroke using methylation levels of 9 genes (*AMH*, *C17orf82*, *HDAC9*, *IGFBP3*, *LRRC10B*, *PDE3A*, *PRDM6*, *SYT7* and *TBX2*).





**Supplementary figure S4 Results for the association between DNA methylations and prognosis of ischemic stroke**

The adjusted *P* values for the association between DNA methylations and modified Rankin Scale score (red dot) and death (green triangles) at 3 month of onset. The associations were adjusted for age, sex, admission NIHSS score, SBP, eGFR, current smoking, alcohol drinking, use of antihypertensive medications, family history of stroke, ischemic stroke subtype, randomized treatment, and history of hypertension, hyperlipidemia, diabetes mellitus, and coronary heart disease. The x axis represents the chromosome and the dots were arranged by their genomic positions (GRCh37.p13). The y axis shows the –log_10_*P* values. A total of 377 methylation sites were analyzed, and 12 of them passed the significance threshold of 1.30 × 10^-4^ (red line). The associated methylation sites tend to cluster in targets.


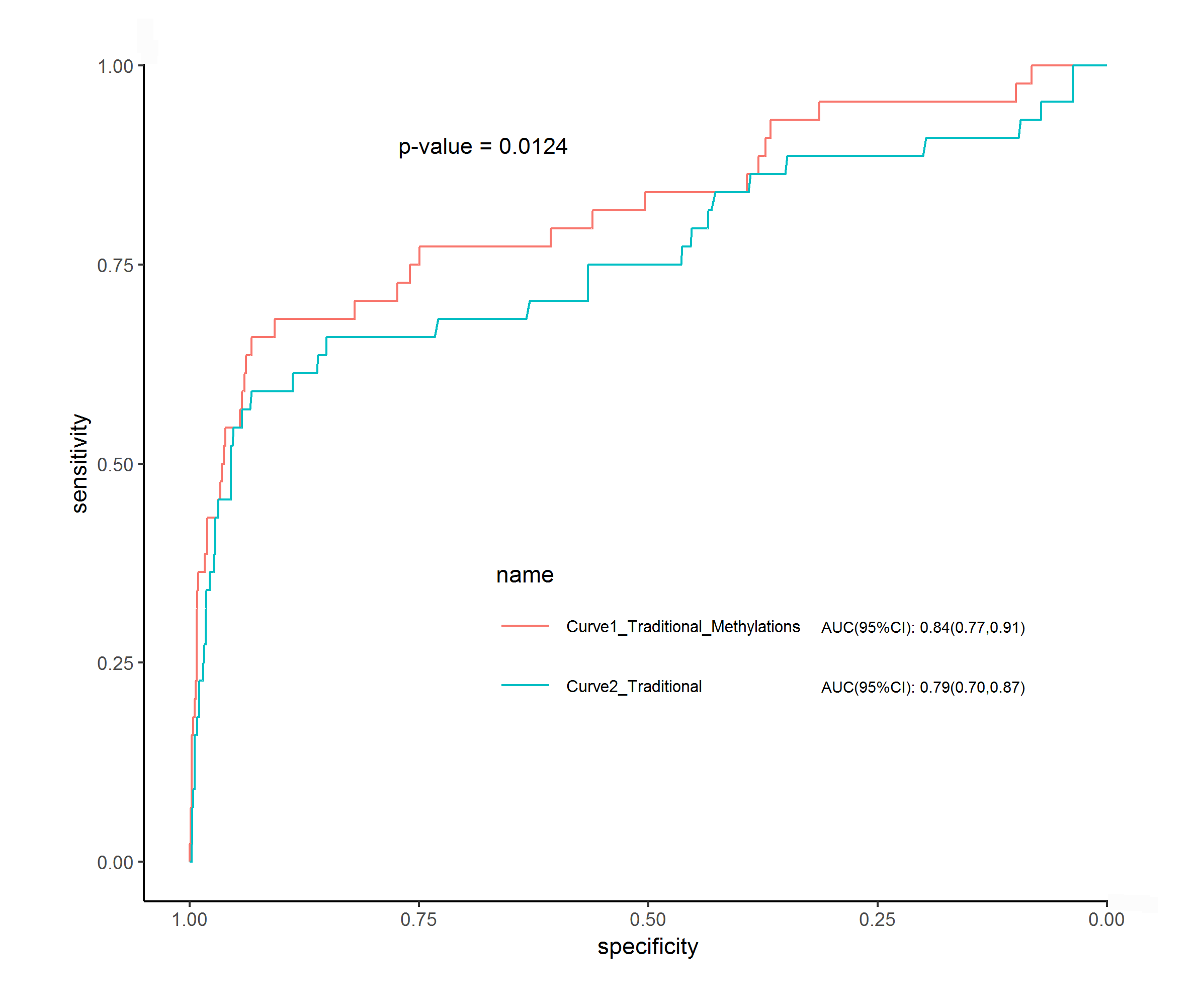


**Supplementary figure S5 ROC for ischemic stroke prognosis prediction in Chinese individuals**

Traditional model admission NIHSS score and age. Methylations included the methylation levels of targets of AMH_3, C17orf82_5, PRDM6_2 and TBX2_3.


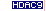

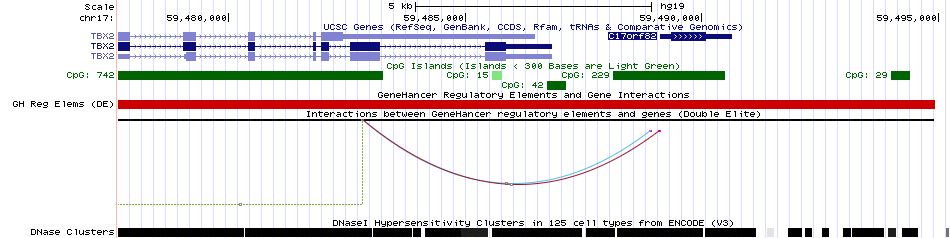

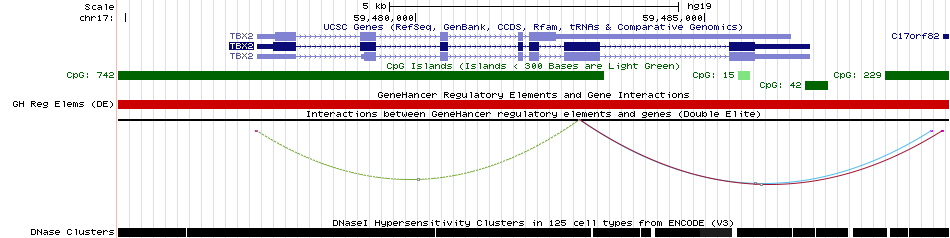

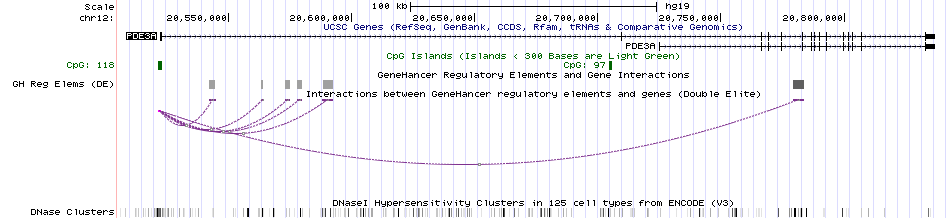

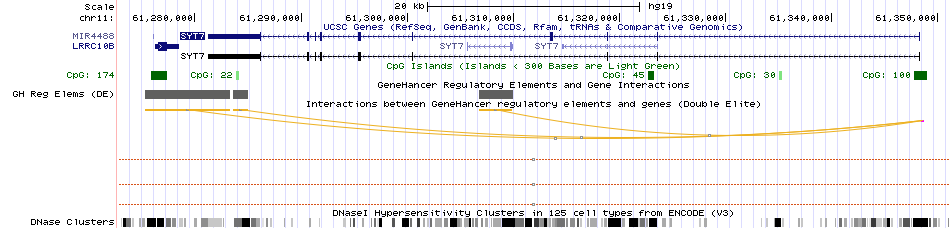

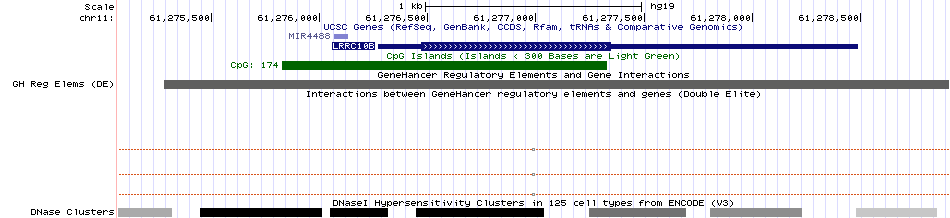

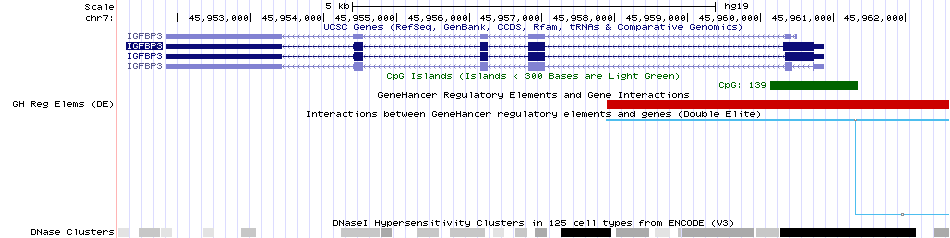

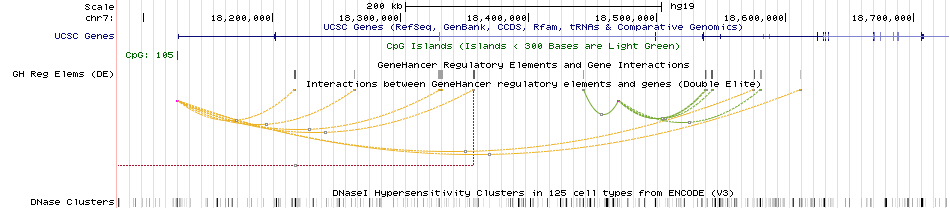

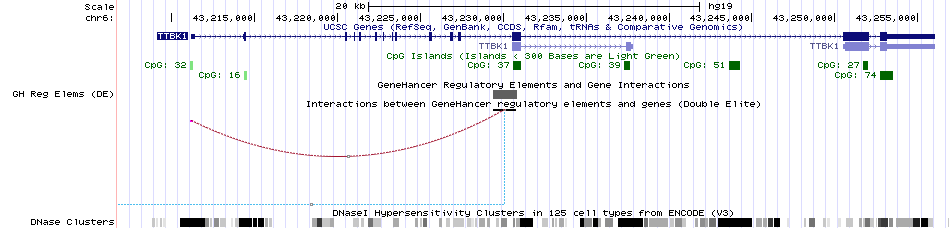

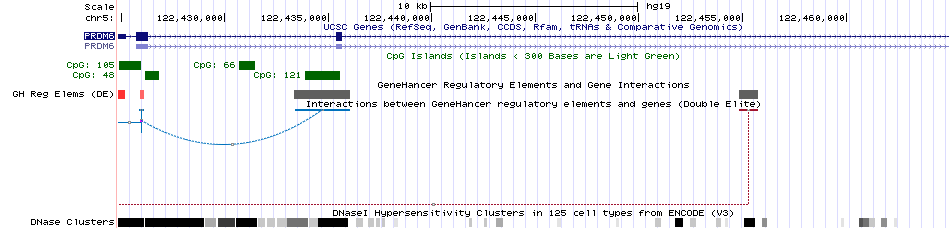

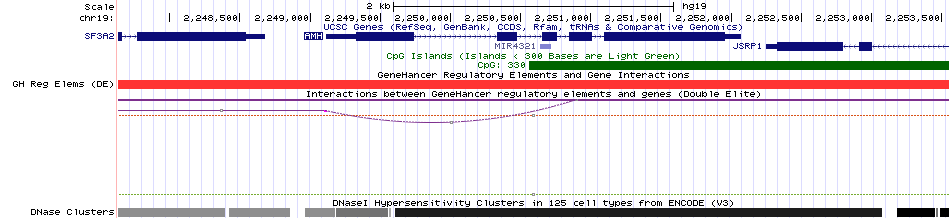


**Supplementary figure S6 Genomic region of the ten newly identified ischemic stroke-associated gene**

The CpG islands tested (red arrows) overlap GeneHancer regulatory elements. In the track named “Enhancers and promoters from GeneHancer”, red and gray colors represent promoters and enhancers, respectively. The track “Interactions between GeneHancer regulatory elements and genes” shows GeneHancers and their targets connected by curves.


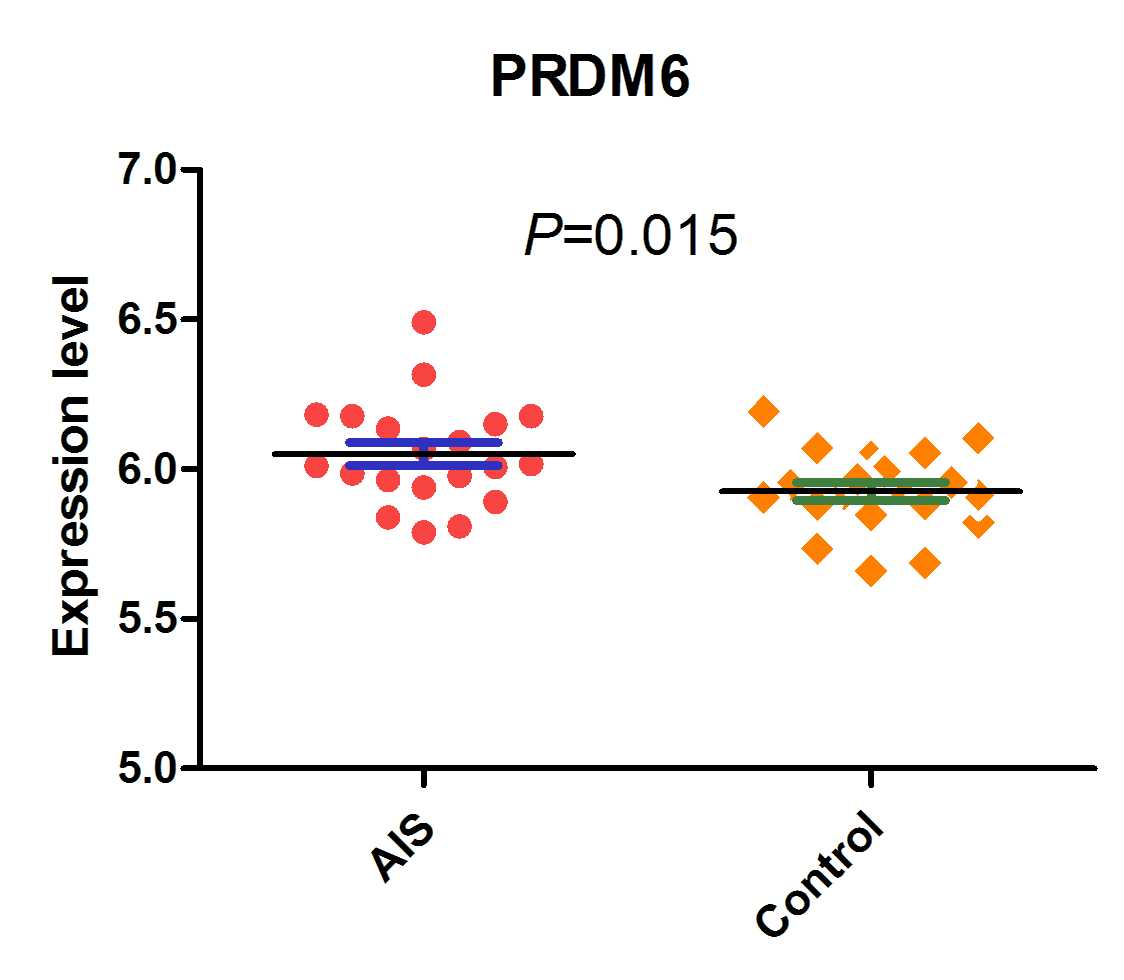


**Supplementary figure S7 *PRDM6* was differentially expressed between ischemic stroke cases and controls**

The data was obtained from a GEO dataset (GSE22255, https://www.ncbi.nlm.nih.gov/gds/). The expression levels were tested in peripheral blood mononuclear cells.
