# Supplementary material for: Association of DNA Methylation in Blood Pressure-Related Genes With Ischemic Stroke Risk and Prognosis
Source: Front Cardiovasc Med. 2022 Mar 8;9:796245. doi: 10.3389/fcvm.2022.796245 (PMC8957103; doi:10.3389/fcvm.2022.796245)
Supplement: Supplementary file 3 [file Data_Sheet_3.docx]

Supplementary table S1 Information on the targets sequenced in this study

| Targets | CHR | Start(hg19) | End(hg19) | Length | Distance to TSS | No. of sites† | CpG position(hg19) | CpG count |
| --- | --- | --- | --- | --- | --- | --- | --- | --- |
| OSR1_2 | 2 | 19557406 | 19557651 | 246 | 721 | 13 | 19555790-19558403 | 163 |
| OSR1_1 | 2 | 19558399 | 19558180 | 220 | -27 | 21 | 19555790-19558403 | 163 |
| KCNK3 | 2 | 26915555 | 26915807 | 253 | -25 | 40 | 26915604-26916551 | 95 |
| PRDM6_1 | 5 | 122423265 | 122423488 | 224 | -1532 | 11 | 122424338-122424539 | 21 |
| PRDM6_2 | 5 | 122424583 | 122424419 | 165 | -378 | 12 | 122424338-122424539 | 21 |
| PRDM6_3 | 5 | 122425111 | 122424917 | 195 | 120 | 19 | 122424906-122425958 | 105 |
| PRDM6_4 | 5 | 122425772 | 122425618 | 155 | 821 | 11 | 122424906-122425958 | 105 |
| PRDM6_5 | 5 | 122426218 | 122426384 | 167 | 1421 | 19 | 122426126-122426836 | 48 |
| ABLIM3 | 5 | 148521167 | 148520966 | 202 | -43 | 19 | 148520993-148521467 | 47 |
| TTBK1 | 6 | 43211012 | 43211204 | 193 | -405 | 12 | 43211115-43211345 | 32 |
| ZNF318_1 | 6 | 43336668 | 43336935 | 268 | 246 | 39 | 43336481-43337832 | 141 |
| ZNF318_2 | 6 | 43337000 | 43337153 | 154 | 28 | 17 | 43336481-43337832 | 141 |
| HDAC9_1 | 7 | 18126026 | 18125758 | 269 | -806 | 18 | 18125915-18127009 | 105 |
| HDAC9_2 | 7 | 18126316 | 18126076 | 241 | -488 | 23 | 18125915-18127009 | 105 |
| HDAC9_3 | 7 | 18547095 | 18546843 | 253 | 420279 | 14 | 18546843-18547095 | - |
| IGFBP3_1 | 7 | 45961440 | 45961172 | 269 | -569 | 16 | 45960138-45961347 | 139 |
| IGFBP3_2 | 7 | 45960480 | 45960262 | 219 | 391 | 28 | 45960138-45961347 | 139 |
| IGFBP3_3 | 7 | 45960753 | 45960958 | 206 | -87 | 22 | 45960138-45961347 | 139 |
| LRRC10B_1 | 11 | 61276114 | 61275868 | 247 | -403 | 19 | 61275826-61277329 | 174 |
| LRRC10B_2 | 11 | 61277075 | 61276891 | 185 | 620 | 23 | 61275826-61277329 | 174 |
| SYT7_1 | 11 | 61348388 | 61348165 | 224 | -44 | 31 | 61347809-61349038 | 100 |
| SYT7_2 | 11 | 61348882 | 61349063 | 182 | -719 | 15 | 61347809-61349038 | 100 |
| PDE3A_1 | 12 | 20521690 | 20521926 | 237 | -488 | 22 | 20521617-20523122 | 118 |
| PDE3A_2 | 12 | 20522200 | 20522368 | 169 | 22 | 16 | 20521617-20523122 | 118 |
| PDE3A_3 | 12 | 20522826 | 20523046 | 221 | 648 | 14 | 20521617-20523122 | 118 |
| TBX2_1 | 17 | 59475331 | 59475132 | 200 | -2124 | 13 | 59473061-59483266 | 742 |
| TBX2_2 | 17 | 59475409 | 59475636 | 228 | -1847 | 11 | 59473061-59483266 | 742 |
| TBX2_3 | 17 | 59476620 | 59476394 | 227 | -862 | 22 | 59473061-59483266 | 742 |
| TBX2_4 | 17 | 59477902 | 59477640 | 263 | 384 | 33 | 59473061-59483266 | 742 |
| C17orf82_1 | 17 | 59486647 | 59486915 | 269 | -2464 | 30 | 59488116-59490485 | 229 |
| C17orf82_2 | 17 | 59488288 | 59488091 | 198 | -1020 | 15 | 59488116-59490485 | 229 |
| C17orf82_3 | 17 | 59488839 | 59489012 | 174 | -272 | 15 | 59488116-59490485 | 229 |
| C17orf82_4 | 17 | 59489907 | 59489675 | 233 | 564 | 17 | 59488116-59490485 | 229 |
| C17orf82_5 | 17 | 59490276 | 59490117 | 160 | 1006 | 14 | 59488116-59490485 | 229 |
| DOT1L_1 | 19 | 2162226 | 2162461 | 236 | -1921 | 13 | 2163633-2165603 | 184 |
| DOT1L_2 | 19 | 2163982 | 2164214 | 233 | -165 | 30 | 2163633-2165603 | 184 |
| DOT1L_3 | 19 | 2164368 | 2164563 | 196 | 221 | 22 | 2163633-2165603 | 184 |
| DOT1L_4 | 19 | 2165174 | 2165409 | 236 | 1027 | 27 | 2163633-2165603 | 184 |
| PLEKHJ1_1 | 19 | 2235568 | 2235823 | 256 | 529 | 15 | 2235682-2237102 | 142 |
| PLEKHJ1_2 | 19 | 2235568 | 2235775 | 208 | -1247 | 12 | 2235682-2237102 | 142 |
| SF3A2_1 | 19 | 2236764 | 2236917 | 154 | -565 | 12 | 2235682-2237102 | 142 |
| AMH_1 | 19 | 2250998 | 2250806 | 193 | 1529 | 20 | 2250561-2253959 | 330 |
| AMH_2 | 19 | 2251310 | 2251567 | 258 | 2033 | 30 | 2250561-2253959 | 330 |
| AMH_3 | 19 | 2252568 | 2252326 | 243 | 3049 | 21 | 2250561-2253959 | 330 |

CHR: Chromosome; TSS: Transcription start site.

†: Number of methylation sites tested in the targets.

Supplementary table S2 Primers for the targets sequenced in this study

| PrimerName | Primer |
| --- | --- |
| ABLIM3_F | GATTTTATGAATTTTGGTTTTYGTTTG |
| ABLIM3_R | TCACACCCTAAAACCCAAACAA |
| AMH_1_F | GGGAAGGGTAYGGTGTTTAGTT |
| AMH_1_R | AACCCCCAACCCCTAAAC |
| AMH_2_F | tgttgttgttgttgAGGTTTATTG |
| AMH_2_R | ACAACRCCTTCAAAAACAACAAC |
| AMH_3_F | GGAGGAGAGATTTAAGAAAGAGAGG |
| AMH_3_R | ACTCAATCCCRCCCCTTACCT |
| C17orf82_1_F | AGGTTTAGAGTTTTGGAGGAGTTATT |
| C17orf82_1_R | CAACCTTTTATCCCCRCCTCTC |
| C17orf82_2_F | TTTTTAATGTTTTGTTGAAAGTGAGAAG |
| C17orf82_2_R | TTCTAACTTCCCTACCTCAAACCTAC |
| C17orf82_3_F | GYGTGGTAGGAGGGTTAGTGTTG |
| C17orf82_3_R | CCTAYACTCACACCCTCCATCT |
| C17orf82_4_F | TTYGTGGATTTGTGAGGAGTTG |
| C17orf82_4_R | ACTTAAACTTCCTTTTAAACTCTCCRAA |
| C17orf82_5_F | GGGTTTAGGAGGTYGGAGGT |
| C17orf82_5_R | AAACCTTCTCCCCTACTACCAA |
| DOT1L_1_F | Ggggttttattatgttagttaggatggt |
| DOT1L_1_R | tctctaactcctaatctcaaactcaaa |
| DOT1L_2_F | GGGTTTGTGATTATAAAGAGGGAGT |
| DOT1L_2_R | ACTTCAATCTCAACTCCAACTTCTC |
| DOT1L_3_F | GGAAYGGAGATTTTGGATTTTATTGT |
| DOT1L_3_R | CACRAATTACCCCTTCCRTTCC |
| DOT1L_4_F | TTYGGGGAAAAGTGAAGTAGG |
| DOT1L_4_R | CTCCCTCTCCCATCCACTC |
| HDAC9_1_F | GGTGTTGGGGAGAAGTTAGG |
| HDAC9_1_R | CAACAATCACCATATTCCCTCAC |
| HDAC9_2_F | GGTTTAGGYGTTGGAGATTYGTAGG |
| HDAC9_2_R | CATATCTCRCTTCAAAAATCCCAAAT |
| HDAC9_3_F | GTTTGTAATTTTAGTATTTTGGGAGGT |
| HDAC9_3_R | TCACTCTATCRCCCAAACTAAAATACA |
| IGFBP3_1_F | TGGGGATATAAATAGTTTAGYGGGTGT |
| IGFBP3_1_R | AATCACTCCTAACCAACTCAACAC |
| IGFBP3_2_F | GTTGTGGTTTYGGTTTTYGTTGT |
| IGFBP3_2_R | ACCCAACAACCCCCAAAC |
| IGFBP3_3_F | GGGGTAYGTTGTTTGGTAGGTT |
| IGFBP3_3_R | CCAACCCCCACTCCTAAAC |
| KCNK3_F | GGGTGGTGTTGAAGGGATAG |
| KCNK3_R | CCACCAACAAATAAATAAAAATACACAC |
| LRRC10B_1_F | AAGGYGTTATTTTAGTGGGGTTTT |
| LRRC10B_1_R | CCCCTCAACCCCTCCTTAT |
| LRRC10B_2_F | GGGTGTAGGTYGGGGAAGT |
| LRRC10B_2_R | CTCCAAATAAACRACAACCRACTAC |
| OSR1_1_F | TYGGTGTTAAGGGATGAGTGAG |
| OSR1_1_R | AAACCCACAAACRCCCCTAAAA |
| OSR1_2_F | AGTAGTGTTTGYGTTTATTGTATTGTTTTT |
| OSR1_2_R | CAAAAACAAATACATACCCCAACTC |
| PDE3A_1_F | GGAAGGAGGAGAAGGGAGA |
| PDE3A_1_R | AATATCTTTCACAAATTCTCTACTCCAC |
| PDE3A_2_F | GAAGAGGGTATTTTATATTATGGTAGTGTT |
| PDE3A_2_R | CAAATCTCCCCAACAACCAC |
| PDE3A_3_F | GGTTGGTGTTGAGGTTGAGGT |
| PDE3A_3_R | AATCAACTAAAACCCCAAATACTCC |
| PLEKHJ1_1_F | AGGTGGGAAGGGTGGTGT |
| PLEKHJ1_1_R | TCRAAACCCTACTACTAAAACRCTACAA |
| PLEKHJ1_2_F | TYGTTGGGYGGGTTAGGTATG |
| PLEKHJ1_2_R | CCRCCTCTACTCCTCACCAAAAC |
| PRDM6_1_F | AGTAATGAATTAGGAGAGTGTGAGAAAG |
| PRDM6_1_R | AACACCCAAATTTAAAACAAACAC |
| PRDM6_2_F | TTGGGTTTTAGTTTAGTGTTTTGATTT |
| PRDM6_2_R | TAACACCCAAAAACACTTCAAAAA |
| PRDM6_3_F | GGGAGGAGAGTTTTAGAGATTGTG |
| PRDM6_3_R | CRTATCCAACCRAAARACTACCA |
| PRDM6_4_F | TTGTAGGTAGGTTGGGTTTATTTTGAG |
| PRDM6_4_R | CCAAAATCCCAATAACCCTCTT |
| PRDM6_5_F | TAGAGGAGTTGGATTATTATTTGTATGGT |
| PRDM6_5_R | ACCCRCCTCCCTAACAaacac |
| SF3A2_1_F | AGGTGGGAAGGGTGGTGT |
| SF3A2_1_R | ACACCTTCTCCATCAATAAATAAAAC |
| SYT7_1_F | GGGGTYGTTYGGTTTTATGTTT |
| SYT7_1_R | ACAACAACCCCAAACTAAAACTC |
| SYT7_2_F | GTTTGGTATYGGGTAGGGTYGTAG |
| SYT7_2_R | CTCCTAATTTTTACACACACCAATC |
| TBX2_1_F | GGGAATTTAGGTATTYGTTTAGGAAT |
| TBX2_1_R | AACCCCTAAACCAACTATAAATAAACTAA |
| TBX2_2_F | GAGAGGTTATATTTYGTTTAAGGTGGA |
| TBX2_2_R | CCCAAAACCCTCACAACTCC |
| TBX2_3_F | GATTTTAGGTGGYGGGAGTTGG |
| TBX2_3_R | TCACCTTCCTAACCCCRAAAAT |
| TBX2_4_F | TTTAGTTTGTGGAATTGGTTTTATAGTT |
| TBX2_4_R | CCCTCCTTCTTCCCRACAC |
| TTBK1_F | TGGGGTTGTGTTGGGAGA |
| TTBK1_R | ACATCAACACCAACCCTAACATC |
| ZNF318_1_F | GGGGATGGAGGGAGTTTTG |
| ZNF318_1_R | CCCCRCTCRCCCTCAAAAC |
| ZNF318_2_F | GGGTTTGAGGAGGAGTTAGAGTT |
| ZNF318_2_R | TAAAACRCCCCCRAATCAAAAC |

Supplementary table S3 The associations between methylation levels of each target and ischemic stroke in sub-groups

| Targets | Male | |  | Female | |  | All | |
| --- | --- | --- | --- | --- | --- | --- | --- | --- |
|  | OR(95%CI) | *P* value |  | OR(95%CI) | *P* value |  | OR(95%CI) | *P* value |
| AMH_3 | 0.58(0.53,0.64) | 1.79×10^-26^ |  | 0.73(0.65,0.81) | 8.04×10^-09^ |  | 0.63(0.59,0.68) | 1.59×10^-33^ |
| C17orf82_2 | 0.90(0.73,1.11) | 3.27×10^-01^ |  | 0.87(0.68,1.11) | 2.60×10^-01^ |  | 0.89(0.76,1.05) | 1.68×10^-01^ |
| C17orf82_3 | 0.67(0.59,0.76) | 4.13×10^-10^ |  | 0.69(0.60,0.80) | 5.08×10^-07^ |  | 0.67(0.61,0.74) | 1.79×10^-16^ |
| C17orf82_5 | 0.47(0.37,0.62) | 2.60×10^-08^ |  | 0.63(0.47,0.84) | 1.72×10^-03^ |  | 0.53(0.44,0.65) | 2.82×10^-10^ |
| HDAC9_1 | 0.62(0.56,0.69) | 2.39×10^-17^ |  | 0.63(0.54,0.73) | 1.34×10^-09^ |  | 0.62(0.57,0.68) | 2.12×10^-25^ |
| IGFBP3_2 | 0.59(0.53,0.66) | 1.55×10^-19^ |  | 0.75(0.67,0.83) | 1.02×10^-07^ |  | 0.66(0.61,0.71) | 1.04×10^-25^ |
| IGFBP3_3 | 0.68(0.61,0.75) | 2.14×10^-12^ |  | 0.79(0.69,0.92) | 2.04×10^-03^ |  | 0.71(0.65,0.77) | 2.71×10^-14^ |
| LRRC10B_1 | 0.49(0.39,0.61) | 1.77×10^-10^ |  | 0.52(0.40,0.68) | 2.45×10^-06^ |  | 0.49(0.41,0.58) | 2.95×10^-16^ |
| LRRC10B_2 | 0.66(0.57,0.78) | 5.39×10^-07^ |  | 0.74(0.62,0.88) | 7.55×10^-04^ |  | 0.69(0.61,0.78) | 1.36×10^-09^ |
| PDE3A_1 | 0.64(0.59,0.70) | 1.52×10^-22^ |  | 0.75(0.68,0.83) | 6.19×10^-08^ |  | 0.68(0.64,0.73) | 7.60×10^-28^ |
| PDE3A_2 | 0.63(0.58,0.69) | 3.22×10^-25^ |  | 0.70(0.63,0.77) | 4.58×10^-12^ |  | 0.65(0.61,0.69) | 1.16×10^-36^ |
| PDE3A_3 | 0.54(0.49,0.61) | 1.24×10^-26^ |  | 0.62(0.54,0.71) | 1.31×10^-12^ |  | 0.56(0.52,0.61) | 1.13×10^-38^ |
| PRDM6_2 | 0.52(0.45,0.61) | 2.00×10^-16^ |  | 0.68(0.56,0.81) | 2.87×10^-05^ |  | 0.57(0.51,0.64) | 3.50×10^-20^ |
| PRDM6_3 | 0.54(0.47,0.62) | 2.78×10^-18^ |  | 0.62(0.53,0.72) | 1.69×10^-09^ |  | 0.56(0.50,0.62) | 1.57×10^-27^ |
| PRDM6_4 | 0.33(0.26,0.42) | 4.85×10^-18^ |  | 0.45(0.34,0.60) | 3.76×10^-08^ |  | 0.36(0.30,0.43) | 1.58×10^-25^ |
| PRDM6_5 | 0.68(0.62,0.75) | 6.51×10^-16^ |  | 0.80(0.72,0.89) | 5.92×10^-05^ |  | 0.72(0.67,0.77) | 3.54×10^-20^ |
| SYT7_1 | 0.27(0.21,0.33) | 4.14×10^-29^ |  | 0.36(0.28,0.46) | 5.10×10^-15^ |  | 0.29(0.25,0.34) | 2.32×10^-44^ |
| TBX2_1 | 0.96(0.93,0.99) | 4.17×10^-03^ |  | 1.00(0.97,1.04) | 8.18×10^-01^ |  | 0.98(0.96,1.00) | 2.28×10^-02^ |
| TBX2_2 | 0.89(0.84,0.95) | 1.58×10^-04^ |  | 0.91(0.86,0.98) | 7.42×10^-03^ |  | 0.90(0.86,0.94) | 8.54×10^-06^ |
| TBX2_3 | 0.57(0.49,0.66) | 1.47×10^-13^ |  | 0.61(0.51,0.71) | 2.02×10^-09^ |  | 0.57(0.51,0.64) | 4.08×10^-22^ |
| TTBK1 | 1.05(0.97,1.13) | 2.37×10^-01^ |  | 1.11(1.02,1.21) | 1.45×10^-02^ |  | 1.07(1.01,1.14) | 1.48×10^-02^ |

ORs and 95% CIs per 5% increase in methylation levels were calculated. The associations were adjusted for age, sex, smoking, drinking, BMI, hypertension, type 2 diabetes, TC, TG, HDL-C and LDL-C.

Supplementary table S4 Reclassification of ischemic stroke by conventional factors and blood cell DNA methylations

| Models | Continuous NRI | |  | IDI | |
| --- | --- | --- | --- | --- | --- |
|  | Estimate (95% CI), % | *P* value |  | Estimate (95% CI), % | *P* value |
| Conventional factors | Reference | — |  | Reference | — |
| Conventional factors + AMH_3 | 53.21(45.45-60.97) | 3.54×10^-41^ |  | 3.35(2.61-4.09) | 7.12×10^-19^ |
| Conventional factors + C17orf82_3 | 21.31(13.35-29.26) | 1.54×10^-07^ |  | 1.13(0.69-1.57) | 4.81×10^-07^ |
| Conventional factors + C17orf82_5 | 19.80(11.78-27.81) | 1.31×10^-06^ |  | 0.62(0.27-0.96) | 4.30×10^-04^ |
| Conventional factors + HDAC9_1 | 45.37(37.51-53.22) | 1.12×10^-29^ |  | 2.45(1.80-3.10) | 1.49×10^-13^ |
| Conventional factors + IGFBP3_2 | 37.59(29.69-45.49) | 1.10×10^-20^ |  | 2.00(1.43-2.58) | 6.10×10^-12^ |
| Conventional factors + IGFBP3_3 | 31.40(23.44-39.37) | 1.06×10^-14^ |  | 1.52(1.02-2.01) | 2.55×10^-09^ |
| Conventional factors + LRRC10B_1 | 30.04(22.08-37.99) | 1.40×10^-13^ |  | 1.21(0.75-1.66) | 2.53×10^-07^ |
| Conventional factors + LRRC10B_2 | 48.18(40.27-56.10) | 7.46×10^-33^ |  | 2.40(1.80-3.00) | 4.51×10^-15^ |
| Conventional factors + PDE3A_1 | 48.93(41.02-56.84) | 7.86×10^-34^ |  | 2.70(2.06-3.34) | 1.35×10^-16^ |
| Conventional factors + PDE3A_2 | 51.44(43.66-59.23) | 2.09×10^-38^ |  | 3.64(2.89-4.39) | 1.86×10^-21^ |
| Conventional factors + PDE3A_3 | 54.56(46.81-62.32) | 2.61×10^-43^ |  | 3.78(3.02-4.55) | 1.87×10^-22^ |
| Conventional factors + PRDM6_2 | 38.16(30.20-46.11) | 5.66×10^-21^ |  | 1.87(1.32-2.42) | 2.67×10^-11^ |
| Conventional factors + PRDM6_3 | 43.40(35.51-51.30) | 4.22×10^-27^ |  | 2.69(2.04-3.34) | 5.01×10^-16^ |
| Conventional factors + PRDM6_4 | 35.76(27.85-43.66) | 7.94×10^-19^ |  | 1.96(1.38-2.54) | 3.51×10^-11^ |
| Conventional factors + PRDM6_5 | 29.55(21.61-37.49) | 3.00×10^-13^ |  | 1.41(0.91-1.92) | 3.25×10^-08^ |
| Conventional factors + SYT7_1 | 59.91(52.21-67.62) | 1.65×10^-52^ |  | 4.09(3.30-4.89) | 3.41×10^-24^ |
| Conventional factors + TBX2_2 | 16.00(7.88-24.12) | 1.10×10^-04^ |  | 0.43(0.14-0.72) | 4.06×10^-03^ |
| Conventional factors + TBX2_3 | 42.82(34.96-50.68) | 1.29×10^-26^ |  | 2.37(1.75-3.00) | 6.77×10^-14^ |

Abbreviations: CI = confidence interval; IDI = integrated discrimination index; NRI = net reclassification improvement.

Conventional model included age, sex, smoking, drinking, BMI, SBP, DBP, GLU and TC.

Supplementary table S5 Associations between methylation levels and major disability and death at 3 months of onset

| Methylation targets | Rankin score | |  | Death or major disability | |  | Major disability (n = 339) | |  | Death (n = 44) | | | |
| --- | --- | --- | --- | --- | --- | --- | --- | --- | --- | --- | --- | --- | --- |
|  | Beta | *P* value |  | OR(95%CI) | *P* value |  | OR(95%CI) | *P* value |  | OR(95%CI) | *P* value | HR(95%CI) | *P* value |
| AMH_3 | -0.12 | **6.97×10**^-04^ |  | 0.97(0.95,0.99) | 5.06×10^-03^ |  | 0.97(0.95,1.00) | 2.35×10^-02^ |  | 0.97(0.96,0.99) | **4.25×10**^-04^ | 0.43(0.27,0.71) | **8.23×10**^-04^ |
| C17orf82_2 | -0.07 | 2.97×10^-01^ |  | 0.97(0.92,1.01) | 1.07×10^-01^ |  | 0.97(0.93,1.01) | 1.59×10^-01^ |  | 0.97(0.94,0.99) | 7.87×10^-03^ | 0.37(0.17,0.80) | 1.09×10^-02^ |
| C17orf82_3 | -0.07 | 1.34×10^-01^ |  | 0.98(0.96,1.01) | 2.75×10^-01^ |  | 0.99(0.96,1.02) | 4.24×10^-01^ |  | 0.98(0.96,1.00) | 2.34×10^-02^ | 0.45(0.25,0.79) | 5.75×10^-03^ |
| C17orf82_5 | -0.28 | 2.77×10^-03^ |  | 0.94(0.88,0.99) | 3.03×10^-02^ |  | 0.95(0.90,1.01) | 9.45×10^-02^ |  | 0.94(0.91,0.98) | **1.34×10**^-03^ | 0.45(0.29,0.70) | **6.38×10**^-04^ |
| HDAC9_1 | -0.14 | **2.40×10**^-03^ |  | 0.96(0.94,0.99) | 1.51×10^-02^ |  | 0.97(0.94,1.00) | 5.27×10^-02^ |  | 0.98(0.96,0.99) | 9.73×10^-03^ | 0.52(0.30,0.91) | 2.15×10^-02^ |
| IGFBP3_2 | -0.12 | 5.16×10^-03^ |  | 0.97(0.94,1.00) | 2.14×10^-02^ |  | 0.98(0.95,1.00) | 9.26×10^-02^ |  | 0.99(0.97,1.00) | 1.26×10^-01^ | 0.57(0.34,0.94) | 2.69×10^-02^ |
| IGFBP3_3 | -0.16 | **4.30×10**^-04^ |  | 0.96(0.93,0.98) | 2.54×10^-03^ |  | 0.96(0.93,0.99) | 7.19×10^-03^ |  | 0.98(0.96,1.00) | 5.29×10^-02^ | 0.55(0.31,0.97) | 3.87×10^-02^ |
| LRRC10B_1 | -0.22 | 4.40×10^-03^ |  | 0.93(0.89,0.98) | 5.86×10^-03^ |  | 0.94(0.90,0.99) | 1.93×10^-02^ |  | 0.97(0.94,1.00) | 9.76×10^-02^ | 0.52(0.21,1.26) | 1.47×10^-01^ |
| LRRC10B_2 | 0.13 | 4.85×10^-01^ |  | 1.05(0.94,1.19) | 3.84×10^-01^ |  | 1.06(0.94,1.19) | 3.29×10^-01^ |  | 0.98(0.91,1.05) | 6.11×10^-01^ | 0.68(0.11,4.19) | 6.76×10^-01^ |
| PDE3A_1 | -0.03 | 3.65×10^-01^ |  | 1.00 (0.98,1.02) | 7.31×10^-01^ |  | 1.00 (0.98,1.02) | 8.49×10^-01^ |  | 0.99(0.98,1.00) | 6.14×10^-02^ | 0.92(0.63,1.35) | 6.87×10^-01^ |
| PDE3A_2 | -0.13 | **1.67×10**^-04^ |  | 0.97(0.94,0.99) | **1.73×10**^-03^ |  | 0.97(0.95,0.99) | 7.58×10^-03^ |  | 0.98(0.97,1.00) | 8.03×10^-03^ | 0.50(0.30,0.82) | 6.46×10^-03^ |
| PDE3A_3 | -0.12 | 3.02×10^-03^ |  | 0.97(0.94,0.99) | 1.03×10^-02^ |  | 0.97(0.94,1.00) | 2.80×10^-02^ |  | 0.98(0.96,0.99) | 3.10×10^-03^ | 0.44(0.25,0.80) | 6.67×10^-03^ |
| PRDM6_2 | -0.24 | **2.58×10**^-05^ |  | 0.95(0.92,0.99) | 8.08×10^-03^ |  | 0.96(0.92,0.99) | 2.40×10^-02^ |  | 0.97(0.94,0.99) | **2.27×10**^-03^ | 0.39(0.21,0.74) | 4.10×10^-03^ |
| PRDM6_3 | -0.17 | **1.10×10**^-03^ |  | 0.96(0.93,1.00) | 3.42×10^-02^ |  | 0.97(0.94,1.01) | 1.35×10^-01^ |  | 0.97(0.95,0.99) | 5.95×10^-03^ | 0.32(0.16,0.63) | **1.06×10**^-03^ |
| PRDM6_4 | -0.17 | 4.91×10^-02^ |  | 0.94(0.89,0.99) | 2.37×10^-02^ |  | 0.95(0.90,1.00) | 6.32×10^-02^ |  | 0.97(0.93,1.00) | 5.75×10^-02^ | 0.33(0.12,0.89) | 2.93×10^-02^ |
| PRDM6_5 | -0.05 | 1.20×10^-01^ |  | 0.99(0.97,1.01) | 4.00×10^-01^ |  | 0.99(0.97,1.02) | 6.20×10^-01^ |  | 0.98(0.97,1.00) | 1.52×10^-02^ | 0.59(0.39,0.90) | 1.36×10^-02^ |
| SYT7_1 | -0.24 | 5.04×10^-03^ |  | 0.95(0.90,1.00) | 4.93×10^-02^ |  | 0.96(0.91,1.01) | 1.31×10^-01^ |  | 0.97(0.93,1.00) | 4.07×10^-02^ | 0.47(0.25,0.87) | 1.65×10^-02^ |
| TBX2_1 | -0.02 | 3.96×10^-02^ |  | 1.00 (0.99,1.00) | 1.75×10^-01^ |  | 1.00 (0.99,1.00) | 3.85×10^-01^ |  | 1.00 (0.99,1.00) | 2.76×10^-01^ | 0.93(0.86,1.01) | 9.32×10^-02^ |
| TBX2_2 | -0.03 | 9.63×10^-02^ |  | 0.99(0.98,1.01) | 3.24×10^-01^ |  | 1.00 (0.99,1.01) | 5.31×10^-01^ |  | 0.99(0.99,1.00) | 1.16×10^-01^ | 0.83(0.70,0.99) | 3.67×10^-02^ |
| TBX2_3 | -0.13 | 1.36×10^-02^ |  | 0.97(0.94,1.00) | 6.77×10^-02^ |  | 0.97(0.94,1.01) | 1.50×10^-01^ |  | 0.96(0.94,0.98) | **3.13×10**^-04^ | 0.61(0.49,0.76) | **3.08×10**^-05^ |
| TTBK1 | 0.02 | 2.69×10^-01^ |  | 1.01(1.00,1.03) | 7.52×10^-02^ |  | 1.01(1.00,1.03) | 6.87×10^-02^ |  | 1.00 (0.99,1.01) | 9.08×10^-01^ | 1.04(0.89,1.22) | 6.29×10^-01^ |

*P* values less than 2.38 × 10^-3^ were highlighted in bold. The associations were adjusted for age, sex, admission NIHSS score, SBP, eGFR, current smoking, alcohol drinking, use of antihypertensive medications, family history of stroke, ischemic stroke subtype, randomized treatment, and history of hypertension, hyperlipidemia, diabetes mellitus, and coronary heart disease.

Supplementary table S6 Reclassification of death at 3 months of onset by conventional factors and blood cell DNA methylations

| Models | Continuous NRI | |  | IDI | |
| --- | --- | --- | --- | --- | --- |
|  | Estimate (95% CI), % | *P* value |  | Estimate (95% CI), % | *P* value |
| Conventional factors | Reference | — |  | Reference | — |
| Conventional factors + AMH_3 | 46.16 (15.97,76.36) | 2.73×10^-03^ |  | 2.58 (0.75,4.41) | 5.77×10^-03^ |
| Conventional factors + C17orf82_5 | 58.25 (28.60,87.90) | 1.20×10^-04^ |  | 2.93 (0.59,5.26) | 1.39×10^-02^ |
| Conventional factors + PRDM6_2 | 49.43 (19.76,79.10) | 1.09×10^-03^ |  | 2.59 (0.62,4.56) | 9.84×10^-03^ |
| Conventional factors + TBX2_3 | 68.10 (41.88,94.31) | 3.57×10^-07^ |  | 3.68 (1.60,5.76) | 5.25×10^-04^ |
| Conventional factors + Combine | 71.57 (43.25,99.89) | 7.30×10^-07^ |  | 6.36 (3.19,9.52) | 8.00×10^-05^ |

Abbreviations: CI = confidence interval; IDI = integrated discrimination index; NRI = net reclassification improvement.

Comparison regarded discrimination performance between multivariate-adjusted models with and without methylations of each target. Conventional model included age, sex, admission NIHSS score, SBP, eGFR, current smoking, alcohol drinking, use of antihypertensive medications, family history of stroke, ischemic stroke subtype, randomized treatment, and history of hypertension, hyperlipidemia, diabetes mellitus, and coronary heart disease.

Supplementary table S7 Functional annotation clustering of the ischemic stroke risk and prognosis-associated genes

| **Biological processes** | **Genes** | ***P* value** | **FDR** |
| --- | --- | --- | --- |
| regulation of developmental process | *IGFBP3, PRDM6, PDE3A, AMH, HDAC9, TBX2* | 3.30E-05 | 1.90E-02 |
| muscle cell differentiation | *IGFBP3, PRDM6, HDAC9, TBX2* | 9.60E-05 | 2.80E-02 |
| muscle structure development | *IGFBP3, PRDM6, HDAC9, TBX2* | 4.10E-04 | 7.10E-02 |
| cell differentiation | *IGFBP3, PRDM6, PDE3A, AMH, HDAC9, TBX2* | 4.90E-04 | 7.10E-02 |
| cellular developmental process | *IGFBP3, PRDM6, PDE3A, AMH, HDAC9, TBX2* | 7.60E-04 | 8.90E-02 |
| regulation of muscle cell differentiation | *IGFBP3, PRDM6, HDAC9* | 9.50E-04 | 9.40E-02 |
